
JOURNAL INFORMATION
==============================
NLM Title Abbreviation: J Orthop Traumatol
Journal ID: J Orthop Traumatol
NLM Journal ID: 101090931

Journal of Orthopaedics and Traumatology : Official Journal of the Italian Society of Orthopaedics and Traumatology

ISSN: 1590-9921
EISSN: 1590-9999
Publisher: Springer

ARTICLE INFORMATION
==============================
PMCID: PMC4417755
DOI: 10.1007/s10195-012-0211-1
Article ID: 211
Article version: 1
Subjects: 13 November 2012

In-Depth Oral Presentations, Oral Communications and Residents’ Oral Communications

Electronic publication date: 2012 Aug 30
Print publication date: 2012 Nov
Volume: 13
Issue: Suppl 1
First page: 91
Last page: 123
Copyright: © The Author(s) 2012
License: Open AccessThis article is distributed under the terms of the Creative Commons Attribution 2.0 International License (https://creativecommons.org/licenses/by/2.0), which permits unrestricted use, distribution, and reproduction in any medium, provided the original work is properly cited.
License URL: https://creativecommons.org/licenses/by/2.0/

issue-copyright-statement: © The Author(s) 2012

==============================
J Orthopaed Traumatol (2012) 13 (Suppl 1):S91–S123
DOI 10.1007/s10195-012-0211-1

13 NOVEMBER 2012

### In-Depth Oral Presentations, Oral Communications and Residents' Oral Communications

### IN-DEPTH ORAL PRESENTATIONS

### *AT09—ARTHROSCOPY*

#### Hip arthroscopy in painful hip arthroplasty

D. Dallari*, C. Stagni, M. Filanti, C. Carubbi, N. Rani

Istituto Ortopedico Rizzoli (Bologna, IT)

**Introduction** In recent years hip arthroscopy is an increasingly popular surgical technique; in patients with painful hip arthroplasty is applicable for diagnostic and therapeutic purposes.
**Materials and methods** From 2009 to 2011, 11 patients with painful hip arthroplasty underwent hip arthroscopy. There were 10 total hip arthroplasty, 1 resurfacing and 1 revision arthroplasty. In all cases there was persistent pain (mean duration of 10.8 months after prosthetic surgery). Exclusion criteria were septic or aseptic prosthetic loosening. In 9 cases, testing was performed with ultrasound-guided infiltration of local anaesthetic, with temporary benefit in 7 cases, confirming the clinical suspicion of iliopsoas tendon impingement.
**Results** Of the 7 patients who underwent arthroscopic iliopsoas tenotomy, 6 showed very satisfactory result, 1 patient showed partial improvement. In 4 cases, intraarticular scar tissue was excised, associated in 2 cases with marked synovitis. The result was good in 2 cases, in the remaining cases a non-significant clinical change was obtained. In 1 case we recorded an intraoperative breakage of arthroscopic instrument, with immediate conversion to open procedure.
**Discussion** The indications for hip arthroscopy are still evolving. We are now oriented to treat patients suffering from ileopsoas impingement, a situation favoured by the use of large cups placed in reduced anteversion or in case of insufficient head/neck ratio in resurfacing. Synovitis or marked joint fibrosis may represent indications for arthroscopic treatment, although with less predictable clinical results.
**Conclusions** Arthroscopy in painful hip arthroplasty is therapeutic in cases of painful psoas tendon, can be considered a valuable diagnostic tool and therapeutic technique in cases of reactive synovitis and arthrofibrosis, where the clinical examination and instrumental exclude mechanical failure or prosthetic loosening.

#### What are the risk factors for recurrent shoulder dislocation arthroscopically managed with absorbable knotless anchors? A statistical analysis

M. Ciccarelli*[1], R. Russo[1], F. Cautiero[1], G. Della Rotonda[2]

[1]U.O.C. di Ortopedia e Traumatologia, Ospedale dei Pellegrini (Naples, IT);
[2]Scuola di Specializzazione in Ortopedia, Università Federico II (Naples, IT)

**Introduction** The purpose of this study was to evaluate the clinical outcome and risk factors for recurrent dislocation after arthroscopic anterior capsulolabral reconstruction with absorbable knotless anchor in a large series of patients with a mean follow-up of 5.6 years.
**Materials and methods** From September 2001 to December 2007 we treated 197 patients with anterior shoulder instability, either traumatic (n = 117) or non traumatic (n = 80). All patients were assessed with the same preoperative protocol and were treated with the same arthroscopic technique. We recorded age at surgery, and number and type of dislocations (traumatic\no traumatic). 129/197 (65.4 %) were re-examined at a minimum 24-month follow-up using either the Rowe scale (n = 81) or the Simple Shoulder Test (SST n = 47).
**Results** In our series the mean Rowe score was 90.8 (range 50–100), and the mean SST was 10.9 (range 8–12); 10/129 patients (7.7 %) had a recurrence. The recurrent dislocation was atraumatic in 4 cases, which reduces the real recurrence rate to 3.1 %. Patients with recurrence were significantly younger at surgery than patients without recurrence ($p = 0.040$). Patients who redislocated were significantly younger at surgery than patients who did not relapse ($p = 0.040$). The probability of recurrence was not significantly affected by either the number ($p = 0.798$) or the type of shoulder instability ($p = 0.751$), or by the amount of glenoid bone loss ($p = 0.184$). We did not observe any instances of synovitis or anchor mobilizations, and only one case of chondral damage (0.07 %).
**Discussion** In the literature there are many case studies that show the repair capsule-arthroscopic labral with anchors without node absorbable, while more frequent use of metal anchors.
**Conclusions** Absorbable knotless suture anchor repair of capsulolabral lesions results in a good outcome (recurrence = 7.7 %) in patients with monodirectional anterior shoulder instability. The only significant risk factor is age younger than 20 years at surgery ($p = 0.040$). Neither the number nor the type of shoulder instability, nor the use of absorbable knotless anchors increases the risk of recurrence.

#### Recurrences after Latarjet: role of arthroscopy

S. De Giorgi*[1], A. Castagna[2]

[1]Università degli Studi di Bari (Bari, IT);
[2]Istituto Clinico Humanitas (Rozzano, Milan, IT)

**Introduction** Many techniques have been proposed for the treatment of shoulder instability. Among these, the Latarjet procedure is one of the most used because it gives good long-term results considering the low percentage of recurrence. However potential complications such as gleno-humeral arthrosis, absorption of the bone block, breakage or malpositioning of the screws, infections or neurological complications can be serious. Moreover, as a result of other traumas, the shoulder can become again painful and unstable. In such cases, the question arises of which technique to employ, since the Latarjet procedure determines substantial subversion of gleno-humeral anatomy. Aim of our study was the analysis of arthroscopic treatment after failure of a Latarjet procedure and to describe the related definitive results.

**Materials and methods** During the period between January 2000 and June 2007, we treated 17 patients (18 shoulders) using arthroscopy, following failure of an open Latarjet procedure. Clinical revision according to the Constant Score, UCLA, ASES, ROWE and the VAS scale for pain, was carried out at a final follow-up of 5 years and 9 months (min 2 years–max 9 years) from latest surgery.

**Results** At final follow-up, the mean Constant score was 78.4/100, the UCLA indicated 27.2/35, the ASES 99.6/120, the Rowe 75.2/100, the VAS 2.9/10. The total incidence of relapse following last surgery (considering both dislocations and subluxations) was 16.7 %. At final follow-up, 1 subject dislocated for a mild trauma (5.6 %), while subluxations or sprains continued in two shoulders (11.1 %). In 11 cases (61 %) return to sport was achieved.

**Discussion** Risk factors for postsurgical recurrence are young age of patients, hyperlaxity, huge bone deficit, contact sports, revision surgeries and the poor quality of soft tissue, new traumas and surgical errors. Gleno-humeral lesions seem to be produced by a new trauma.

**Conclusions** Suture-anchor arthroscopic technique can in selected cases, lead to satisfactory results, allowing identification and treatment also of intra-articular associated lesions.

### Biomechanical function of anterior cruciate ligament remnants: do they contribute to knee stability?

F. Rosso[1], L. Ravera[2], E. Graziano[3], L. Drocco[3], E.C. Cenna*[3]

[1]II Clinica Ortopedica, Università degli Studi di Torino, CTO-M. Adelaide (Turin, IT);
[2]Università degli Studi di Torino (Turin, IT);
[3]II Clinica Ortopedica, CTO-M. Adelaide (Turin, IT)

**Introduction** Recent studies have reported that human ACL remnants contain several types of mechanoreceptors and that they may contribute to proprioceptor functioning in the knee. Other Authors has already demonstrated that ACL remnants initially contribute to antero-posterior knee stability but they lose their biomechanical function up 1 year after injury. Aim of this study is to demonstrate that ACL remnants can be useful in its reconstructive surgery, and it improves the ligamentation and, overall, early stability of our graft.

**Materials and methods** Our experience is based on three different surgical procedures: (1) Healing Response (HR), ACL femoral insertion microfractures in cases in which ACL has anatomical site but with loss of biomechanical function in selected patients: prepuberty young patients or elder people with lower functional request (2) ACL partial reconstructions (one bundle) (PR) in case of partial rupture of the AM or PL bundle preserving the scar with the bundle remaining anatomically and sometimes functionally active. (3) ACL traditional reconstructions (TR) with trans-tibial technique with hamstrings or patellar tendon preserving as more notch scar tissue as possible. We checked 60 patients with age between 13 and 40 years and follow-up from 1 to 5 years. 20 patients underwent HR, 6 patients PR and 34 patients TR. In all patients more remnants scar tissue as possible was preserved and graduated at time of surgery as covering 25, 50 or at least 75 % of the tendon implant. All the patients were clinically evaluated (with IKDC, KOOS and Lisholm form) and with KT 1000 immediately before surgery and at time of follow-up.

**Results** In HR group in all patients all residual scar was maintained with good or excellent results in more than 80 % of cases. Partial reconstructions and traditional reconstructions preserving as more ACL remnants as possible showed a shorter period of articular inflammation (less swelling) in the first 4 weeks. All the patients begin to swim, run and cycling between 6th and 8th post-operative week.

**Discussion** In 98 % of patients in PR and TR group we had good results, as in the 80 % of HR patients. No complications were found in patients in whom we preserved as more ACL remnants as possible.

**Conclusions** In our experience, ACL remnants contributed to obtain knee stability and allow to the surgeon to use different procedures and to the rehabilitator to adopt more aggressive patterns.

### *AT10—PAEDIATRIC ORTHOPAEDICS*

### Endomedullary osteosynthesis with T.E.N. in metadiaphyseal and diaphyseal femur fractures in children (ESIN). The experience of Orthopaedics and Trauma Unit of A.O.R.N. “Santobono Pausilipon Annunziata” of Naples

P. Di Giacomo, N. Buompane, B. Sorrentino, G. Federico*, M. Traisci, M. Sansò, P. Guida

A.O.R.N. “Santobono Pausilipon Annunziata” (Naples, IT)

**Introduction** Diaphyseal and metaphyseal femur fractures are 1, 6 % of all fractures in children with incidence of 1:5,000. The osteosynthesis with elastic intramedullary nailing (ESIN), according to the indications AAOS, represents the golden standard for reasons including mini-invasive surgery, no need for casting, early mobilization. Nowadays the surgical approach has become prevalent.

**Materials and methods** We performed a retrospective analysis of 62 metadiaphyseal and diaphyseal femur fractures in children age ranged between 3 and 14 years (mean age 8 years) treated with AO techniques during the period 2007–2010. During this 4 years period, of a total of 24,327 admissions, 430 hip fractures (mean 100\year) and of these about 20/year were treated with elastic nailing; treatment with TEN was performed in 62 cases out of 5,668 surgical treatments. The fractures were systematized according to the AO classification. The double access technique using two retrograde nails has been performed in 90 % of cases. In some cases was used the single access side. In metaphyseal fractures a subtrocanteric access with antegrade progression was used. Serial clinical and radiological controls were performed after the first 45\60 days and the next 90 days. The follow-up was on average 3 years.

**Results** Clinical and radiologic union was achieved within 6–8 weeks and coincided with the weight bearing. The recovery of motion of the knee and ankle occurred already after the first 2\3 weeks. Only a small percentage of cases have been applied a cast brace for the first 3 weeks and the full loading was allow after 8 weeks. The return to school was about a week after discharge and the average hospitalization was 3–5 days. The early recovery of function of the knee and ankle has allowed the application only to swimming as a rehabilitation mode.

**Discussion** Compared to fixation with plates or with FEA, treatment with ESIN allows a rapid union of the fracture thanks to an early formation of abundant periosteal callus. This avoids the prolonged immobilization and complications of traction and spica casting.

**Conclusions** The ESIN is a real progress in the treatment of fractures in children: it is a safe technique, minimally invasive, appears to have few complications, does not interfere with growth, and it is associated with rapid return to weight bearing and daily activity.

### Neurological complications of paediatric humeral supracondylar fractures: retrospective analysis of 5,119 cases treated at A.O.R.N. "Santobono Pausilipon Annunziata" of Naples

P. Di Giacomo*[1], G. Federico[1], A. Sorrentino[1], B. Di Maio[1], M. Rizzo[1], L. Carbone[1], N. De Sanctis[2], P. Guida[1]

[1]A.O.R.N. "Santobono Pausilipon Annunziata" (Naples, IT); [2]Campolongo Hospital (Eboli, Salerno, IT)

**Introduction** Humeral supracondylar fractures represent 80 % of all elbow fractures. Neurological complications are present in 10–20 % of cases and include immediate complication, within 8 h of trauma, early complication, post-treatment and late complication, apparent at cast removal. Aim of this study is to realize if predictive factors of complication exist.

**Materials and methods** In the period 1996–2009 we have treated 5,119 Gartland's type III supracondylar fractures (with total loss of contact between the fragments) with reduction and percutaneous trans-lateral condylar fixation with 2–3 Kirschner wires (Judet-Arino Technique).

**Results** In 358 cases (7 %), a neurological complication was diagnosed. In 136 cases anterior interosseous nerve is invoved, with immediate symptoms in 13 cases, early in 113 and late in 10. Radial nerve is involved in 97 cases (27 %), with immediate symptoms in 21 cases, early in 49 and late in 23. Median nerve is involved in 71 patients (19.8 %), with immediate symptoms in 59 cases, early in 5 and late in 7. Ulnar nerve is involved in 54 patients (15 %), with immediate symptoms in 12 cases, early in 35 and late in 7. Electrophysiological studies with VCM and VCS evaluation is done 2 and 4 months after onset. Neurological lesions consist in Seddon's type I (neuropraxia) and type II (axonotmesis) with no type III (neurotmesis) lesions. Nerve function is recovered within 6 months for anterior interosseous nerve; 4–5 months for radial nerve; 6 months for median nerve and 6–7 months for ulnar nerve.

**Discussion** In predicting the neurological sequelae of supracondylar fractures, we must consider: the sharp morphology of fracture fragments with "spade-shaped"; displacing type with anterior interosseous and median nerve damaged in extension-type fractures, radial nerve in antero-lateral displaced fractures and ulnar nerve in flexion-type fractures. Recovery times are 6–7 months. Lateral pinning with 2 k-wires at least and crossing the cortical bone of both sides is preferable.

**Conclusions** Supracondylar fractures related neuropathy tends to a spontaneous healing. So, clinical observation is the initial treatment of choice. In case of persistence of significant symptoms at 6 months after trauma, surgical exploration is indicated.

### *AT11—NEOPLASTIC DISEASES AND INFECTIONS*

### Prion proteins (PRNP and PRND) are over-expressed in osteosarcoma

V. Sollazzo*[1], M. Galasso[2], V. Stefano[3], F. Carinci[4]

[1]Clinica Ortopedica, Università di Ferrara (Ferrara, IT); [2]Data Mining for Analysis of Microarrays, Dept. of Morphology and Embryology, Università degli Studi (Ferrara, IT); [3]Comprehensive Cancer Center, Ohio State University (Columbus, USA); [4]Istituto di Chirurgia Maxillofacciale, Università di Ferrara (Ferrara, IT)

**Introduction** Although osteosarcoma is the most common bone malignancy, the molecular and cellular mechanisms influencing its pathogenesis have remained elusive. Prion proteins (PRNP and PRND), known mostly for its involvement in neurodegenerative spongiform encephalopathies, have been recently demonstrated to be involved in resistance to apoptosis, tumorigenesis, proliferation and metastasis. The main aim of research was to study whether prion proteins were over-expressed in human osteosarcoma, and if prion proteins could have a role also in osteosarcomas.

**Materials and methods** We evaluated differential gene expression between 22 cases of osteosarcoma and 40 cases of normal bone specimens through cDNA microarray analysis spanning a substantial fraction of the human genome.

**Results** PRNP and PRND are significantly over-expressed in osteosarcoma. PRNP and PRND appear involved with some important genes related to tumorigenesis and apoptosis. PRNP is linked to PTK2, RBBP9 and TGFB1 while PRND is linked to TNFSF10, BCL2A1, NFKB2 and TP53RK.

**Discussion** Increased expression on Affymetrix arrays of prion proteins seems to be associated with the development of osteosarcoma. Prions seem to induce a negative regulation of apoptosis, thus promoting osteosarcoma development and progression. Osteosarcoma is a very aggressive tumor and even after modern chemotherapy and excision of tumours efforts are needed to improve clinical outcome.

**Conclusions** Since Prion proteins seem to be related to osteosarcoma development, their inhibition could represent a new approach to the molecular treatment of osteosarcoma.

### Analysis of risk factors of local recurrence in soft tissue sarcomas of the limbs

G. Beltrami*, D. Campanacci, G. Caff, F. Frenos, S. Puccini, G. Scoccianti, R. Capanna

Ortopedia Oncologica e Chirurgia Ricostruttiva, Careggi, CTO (Florence, IT)

**Introduction** Local recurrences (LR) in Soft Tissue Sarcoma (STS) represent a failure of tumour local treatment, and often are related to tumour progression and secondary amputations. Many papers try to focuses the risks factors of recurrences, but there is no general agreement on different variability and prevalence. We report the analysis of 1,055 cases treated in our Centre in the last 20 years, focusing on different risks factors of local recurrences.

**Materials and methods** At Department of Orthopaedic Oncology of CTO-Florence, we have treated, from January 1989 to December 2009, 1,055 patients affected by STS of limbs and superficial trunks. All the patients have been operated and followed by the same equipe of surgeons, radiotherapist, microsurgeons and pathologist, everybody practice in soft tissue sarcoma cases. A surgical intervention of limb salvage was obtained in 972 cases (92 %), while a primary amputation in 83 (8 %). Adjuvant protocols of radio and or chemotherapy was associated to surgery in 907 cases (86 %) and free or rotational flaps in 178 cases (17 %) Local Recurrence Rate (LRR), prevalence and risks factors have been analyzed on the overall group and a statistical analysis have been performed. The risks factors analyzed for LRR were: gender, histotype, site (upper-lower limb and trunk, proximal and distal), size(<5 cm, >5<10 cm, >10 cm), type of presentation (virgin vs. local recurrence previous elsewhere treatment, radicalizations inadequate treatment elsewhere), deepness (superficial-deep muscular fascia), type of surgery (limb salvage vs. amputations), type of surgical margins (radical, wide, marginal, intralesional), primary closure vs. flaps employment, type of flaps(rotational vs. free), type of adjuvant treatment(External Beam Radiotherapy vs. Brachitherapy ± EBRT vs. Chemotherapy), oncological results(continuous disease free, alive with disease, non evident disease, died of disease), functional results (MSTS score).

**Results** At an average follow up of 7 years (1–20), local recurrences occurred in 115 patients (11 %), treated by limb salvage surgery in 87 (76 %) and by secondary amputations in 28 (24 %).The analysis of risks factors showed high grade, histotype like synovial sarcoma or leyomiosarcoma, inadequate surgery, location in superficial trunk, lesion more than 10 cm and surgery without adjuvant therapy as the worst prognostic factors in LRR of STS. In our cases local recurrences were always associated to systemic disease progression, confirming the bad influence on overall survival.

**Discussion** R is a negative event after STS treatment, influenced by histological grade, tumour histotype, site, size and inadequate surgery.

**Conclusions** Adjuvant treatment by radiotherapy is effective in reducing overall LRR. Only a multidisciplinary and super specialized approach may improve the local and systemic prognosis.

### The use of Magnetic Resonance guided Focused Ultrasound for a non-invasive treatment of osteoma osteoid

A. Napoli*, B.C. Marincola, M. Anzidei, M. Mastantuono, O. Moreschini, P. Piciocco, R. Cannata, C. Catalano

Policlinico Umberto I (Rome, IT)

**Introduction** Unlike the other ablative techniques, the Magnetic Resonance guided Focused Ultrasound (MRgFUS) is completely non-invasive, safe and able to ensure a visual check of the treated area in real time.

**Materials and methods** This study was performed on 6 patients, with a mean age of 21 aa, with a clinical-radiographic diagnosis of osteoid osteoma. All patients underwent an ablation with MRgFUS. Exclusion criterion was the location in the vertebral body. The localization of lesions near joints or neurovascular bundles did not represent a contraindication to the use of this method. The clinical success was determined by a clinical follow-up and through the use of imaging techniques to 1–3–6 months; the Visual Analog Pain Score (VAS) was used for the evaluation of the remission of painful symptoms. The treatment was carried out using a variable number of sonication ($4 \pm 1.8$) with an energy of $866 \pm 211$ J.

**Results** A significant difference ($p = 0.001$) between the VAS pre and post surgery was observed ($8.3 \pm 1.6$ and $0.6 \pm 1.5$, respectively). All 6 patients achieved a complete remission of symptoms. There were no complications.

**Discussion** MR guided Focused Ultrasound is a relatively quick technique done in one sitting. RMN guide provides a meticulous overview of the treatment area and adjacent areas for a accurate control by MR thermometry. The latter technique allows monitoring of increased temperature, indirect estimation of thermic damage of periosteum and surrounding tissue. Subsequent imaging studies have documented the progressive and gradual disappearance of oedema and hyperemia typically associated to osteoma osteoid. There wasn't a direct correlation between the disappearance of the nidus and the remission of symptoms. The study with dynamic-ce MR showed the presence of nidus perfusion at 6 months in 4 cases in total absence of pain symptoms.

**Conclusions** The treatment with MRgFUS of osteoid osteoma can be performed successfully with safety without risk of complications.

### *AT12—BIOMATERIALS*

### The use of demineralized bone matrix and mesenchymal concentrated stem cells for the treatment of the simple bone cysts

L. Cevolani*, T. Frisoni, B. Spazzoli, B. Dozza, E. Lucarelli, D.M. Donati, S. Giannini

Istituto Ortopedico Rizzoli (Bologna, IT)

**Introduction** The simple bone cysts are benign lesions that are generally located in the epiphysis of the long bones during growth. The most frequent locations are the humerus and the femur. Cysts are often asymptomatic and frequently healed when skeletal maturity is reached. However, the high risk of pathological fractures justifies the treatment. The aim of the study is to develop a new mini-invasive technique that can reduce the number of hospitalizations needed.

**Materials and methods** The technique consists of a single injection with bone marrow (concentrated using the Cascade® method) and demineralized bone matrix. From November 2007 to February 2011 we treated 66 patients aged 4–37 years (average 12). The mean follow-up was 24 months (6–46 months). The patients were evaluated clinically and radiographically 2, 6 and 24 months after the operation and then every 6 or 12 months. The treatment was evaluated using the radiographic classification by Neer.

**Results** There was a 77 % success in treatment. The 22 % of the cases needed more than one treatment and the 12 % of the cases had a pathological fracture. After 24 months from the operation, Neer's index I or II was presented by 71 % of the patients, Neer III by 22 % and Neer IV by 7 %.

**Discussion** At present, there is not a final treatment for this pathology. The invasive treatment includes the curettage and the application of bone grafts, the multiple perforation of the cystic cavity or the application of cannulated screws of titan or of hydroxyapatite. These techniques are very invasive; the curettage can also cause a growth place damage. Less invasive procedures are the injection with steroids or with bone marrow with or without adjuvant. However, these techniques require multiple hospitalizations and are poorly tolerated by the patients. Our technique allows obtaining very satisfactory results with a single hospitalization.

**Conclusions** Our treatment is efficacious for the therapy of simple bone cysts. This technique can reduce the cost of disease management because of the lows number of hospitalizations needed. This technique cannot reduce the fracture risk.

### One step cartilage repair by PVA-H hydrogel implants in the knee: long-term results

F.V. Sciarretta*, C. Ascani

Clinica Nostra Signora della Mercede (Rome, IT)

**Introduction** Treatment of chondral defects in the knee is actually extremely demanding for orthopaedic surgeons and no "gold standard" has still been determined. This study retrospectively evaluates and presents long term results of PVA-H hydrogel implants in the treatment of knee chondral focal and symptomatic defects.

**Materials and methods** We present clinical and MRI evaluation of 18 patients with III and IV degree knee chondral or osteochodral defects treated by PVA-H hydrogel implants. Defects were no larger than 20 mm in diameter. Average age at time of surgery was 54 years. Patients were 11 male and 7 female. 15 implants were 10 mm and 5 15 mm in diameter. One patient was treated bilaterally.

One patient received two implants. Majority of surgeries have been performed arthroscopically. Average age of implants: 5.9 years (longest: 8 years 3 months; shortest: 4 years). Patients were assessed by IKDC, SF36 scores and MRI imaging.

**Results** All patients showed improvement of knee function and knee scores, in many cases over 50 points of IKDC, except three. Of these, the first one maintained a rather good quality of life for over 5 years with approximately the same level of functionality she enjoys now, after implant removal and knee replacement during 2008. The second case was a 43-year-old female with a post-traumatic chondral defect in a valgus knee. The patient experienced post-op. pain: the implant was removed among another institution at 6 months post-op and was converted to OATS. The third case was a 49 year old male with a severe arthritic pre-op knee and may not have been ideal candidate for Cartiva, but was too young for a total knee replacement at the time of operation and, despite a severe knee worsening during the last year that will need in the short future a knee replacement, has, at over 6-year follow-up, an IKDC score of 33.33 from a pre-op. of 37.93.

**Discussion** The long term follow-up enabled us to conclude that the use of PVA-H implants in knee chondral defects in middle aged patients can guarantee critical knee function improvement and severe pain reduction. Even those patients, who needed a knee replacement, have well done for the first four-five years.

**Conclusions** In our opinion this type of treatment with the correct indications and future implant and instrumentation improvements, already in course, may guarantee a several-years period of knee health and active life style.

### Polyethylene oxidation: can Alpha Lipoic Acid be an alternative to Vitamin E?

F. Conteduca*[1], F. D'angelo[2], J. Conteduca[1], R. Iorio[1], D. Mazza[1], A. Ferretti[1]

[1]Ospedale Sant'Andrea (Rome, IT);
[2]Università La Sapienza (Rome, IT)

**Introduction** Alpha Lipoic Acid (L.A.) is an effective natural antioxidant. It is inside broccoli, spinach and red meats, especially liver and spleen. Actually it is largely used as antioxidant in antiaging products according to the low toxicity level of the product. The present study take into consideration the possibility to reduce oxidation of medical irradiated UHMWPE GUR 1050, mixing together polyethylene powder and Alpha Lipoic Acid powder.

**Materials and methods** The study is composed of two parts. Part 1: thermostability of Alpha Lipoic Acid during polyethylene fusion; Part 2: detection of oxygen level in artificially aged irradiated polyethylene Solid pieces were made with Gur 1050 powder (Ticona Inc., Bayport, Tex, USA) and mixed with 0.1, 0.3, 0.5, 1, 2 % Alpha Lipoic Acid (Talamonti, Italy, Stock 1050919074) and gamma ray irradiated with 30 kGy (Isomedix, Northborough, MA). An oven (80° C) was used to produce an aging effect for 35 days in the doped and control samples (Conventional not doped polyethylene). This process simulates an aging effect of 10 years into the human body. *THERMAL STABILITY* : a Fourier Transfer Infra Red (FTIR) test was made in pieces molded in a cell at 150 and 200 °C and pressure of 200 MPa comparing to the UHMWPE powder mixed with Alpha Lipoic Acid. The presence of Alpha Lipoic Acid in the polyethylene was found at any depth in the manufacts. *OXIDATION*: After 5 weeks at 80° C in an oven (ASTM standard F-2003-02) a Fourier Transfer Infra Red Test (FTIR) was made in the superficial layer and deeper on the undersurface of doped 0.1 % and conventional UHMWPE. The antioxidation limit is defined as the ratio of the area under 1,740 $cm^{-1}$ carbonyl and 1,370 $cm^{-1}$ Methylene absorbance peaks.

**Results** In conventional UHMWPE, oxidation is detected on the surface and decreases in the deeper layers down to zero under 1500 Micron. In the doped UHMWPE, FTIR demonstrate a very low oxidation limit on the surface and at any depth, comparing to conventional UHMWPE.

**Discussion** Our hypothesis is confirmed, Lipoic Acid decreases the UHMWPE oxidation. It's a cheap alternative to Vitamin C. It's natural and so tolerated.

**Conclusions** The examples show that Lipoic Acid is effective as an antioxidant in irradiated UHMWPE and it is stable with respect to thermal treatment.

### Arthroscopic lateral collagen meniscus implant at 2-year minimum follow-up

G.M. Marcheggiani Muccioli[1], A. Grassi*[1], P. Bulgheroni[2], E. Bulgheroni[2], M. Busacca[1], F. Raggi[1], M.P. Neri[1], S. Zaffagnini[1], M. Marcacci[1]

[1]Istituto Ortopedico Rizzoli (Bologna, IT);
[2]Ospedale di Circolo (Varese, IT)

**Introduction** The purpose of this paper is to provide results of the implantation of a bioresorbable type I collagen scaffold projected to restore the meniscal structure (CMI) in patients with irreparable lateral meniscus deficiencies at 2 year minimum follow-up (FU).

**Materials and methods** 24 non-consecutive patients (20 M, 4 F; mean age 35.4 ± 10.76 years, range 16–53) with irreparable lateral meniscus tears or meniscus loss requiring surgical treatment were prospectively enrolled in this Italian multicenter study (2 centers). Patients were evaluated at baseline, at 6 month and 2 at year minimum follow-up (mean 26.7 ± 7.1 month; range 24–32 month) after Lateral CMI implantation, with a 100-point Visual Analogue Scale (VAS) for pain, objective International Knee Documentation Committee (IKDC), Lysholm score, Tegner activity and EQ-5D survey. MRI evaluation was also performed at baseline and at 3 years mean follow-up with both Yulish and Genovese score.

**Results** FU evaluation showed an improving of all the clinical scores: VAS score significantly decreased from 5.5 ± 2.9 to 1.8 ± 1.8 at 6 month FU ($p < 0.0001$) and remained almost stable at 2 year minimum follow-up (1.9 ± 2.5; $p = 0.8259$). Objective IKDC significantly improved from 6A, 14B, 4C at baseline to 20A,3B,1D ($p < 0.0001$) at 6 month FU and did not changed at final FU. Lyhsolm score significantly improved from 64.0 ± 16.2 pre-op to 89.9 ± 11.4 ($p < 0.0001$) at 6 month FU and showed a trend to improve at the final follow-up (92.7 ± 13.8; $p = 0.1218$). Tegner activity Level significantly improved from 3 (2–4) at baseline to 5 (4–7) final follow-up ($p = 0.0062$). The EQ-5D score improved significantly from 0.579 ± 0.28 to 0.892 ± 0.14 at the final FU ($p < 0.0001$). The MRI evaluation showed a trend of the Yulish score to decrease both for lateral tibial plateau (from 1.5; 1.0–2.0 to 1.0; 1.0–0.5; $p = 0.8203$) and for lateral femoral condyle (from 2.0;1.0–2.0 to 1.5;1.0–2.0; $p = 0.9697$). The Genovese score at the final FU was 1.0 (1.0–1.0) for morphology and 1.0 (1.0–2.0) for intensity. No correlation was found between MRI and clinical scores. Neither reoperation nor complications were recorded during the follow-up. Only one patient showed unsatisfactory result.

**Discussion** The obtained results are comparable to those reported for medial CMI at 2 and 5 years of FU, respectively [1, 2].

**Conclusions** Based on available results, with 2 year minimum FU, 96 % of the patients benefited from the Lateral CMI implantation and regained activity and mobility. Longer-term data are being collected to determine the extent and duration of the benefits as observed for Medial CMI [3].

**References**
1. Rodkey et al. (1999) Clin Orthop Rel Res 367(Suppl):S281–S292
2. Bulgheroni et al. (2010) Knee 17:224–229
3. Zaffagnini et al. (2011) Am J Sports Med 39(5):977–985

### Physical, mechanical and pharmacological properties of coloured bone cement with and without antibiotics

O. Galasso[1], G. Calonego[2], A. Corigliano*[1], F. Ranuccio[1], G. Gasparini[1]

[1]Cattedra di Ortopedia e Traumatologia, Università degli Studi "Magna Græcia" di Catanzaro (Catanzaro, IT);
[2]Tecres S.p.A (Verona, IT)

**Introduction** Coloured bone cements have been introduced to make the removal of cement debris easier at the time of primary and revision joint replacement. We evaluated the physical, mechanical and pharmacological effects of adding methylene blue to bone cement with or without antibiotics (gentamicin, vancomycin or both).
**Materials and methods** 8 experimental groups were prepared with different formulation (cement alone or in combination with methylene blue and/or antibiotics). We evaluated the setting time and the maximum polymerisation temperature of the cements. The tests for compressive and bending strength were performed according to the ISO recommendations. Antibiotic concentrations alone and in combination in the eluted samples and the standards were analysed using the standard large plate agar-well diffusion method.
**Results** The addition of methylene blue to plain cement significantly decreased its mean setting time (570 ± 4 s vs. 775 ± 11 s, $p = 0.01$), mean compression strength (95.4 ± 3 MPa vs. 100.1 ± 6 MPa, $p = 0.03$), and mean bending strength (65.2 ± 5 MPa vs. 76.6 ± 4 MPa, $p < 0.001$) as well as its mean elastic modulus (2744 ± 97 MPa vs. 3281 ± 110 MPa, $p < 0.001$). The supplementation of the coloured cement with vancomycin and gentamicin decreased its mean bending resistance (55.7 ± 4 MPa vs. 65.2 ± 5 MPa, $p < 0.001$). The methylene blue significantly decreased the mean release of gentamicin alone (228.2 ± 24 μg vs. 385.5 ± 26 μg, $p < 0.001$) or in combination with vancomycin (498.5 ± 70 μg vs. 613 ± 25 μg, $p = 0.018$) from the bone cement.
**Discussion** Few studies dealing with the use of aqueous methylene blue to make a coloured bone cement with and without antibiotics have been published. We found several differences in physical, mechanical and pharmacological characteristics between the experimental groups. Methylene blue significantly decreased the release of gentamicin alone or in combination with vancomycin from the cement.
**Conclusions** This study demonstrates several theoretical disadvantages of the antibiotic-loaded bone cement coloured with methylene blue.

### *AT13—SHOULDER AND ELBOW*

### The relationship between acromion thickness and body habitus. Practical implications in subacromial decompression procedures

P. Albino*, S. Gumina, D. Passaretti, S. Carbone, V. Arceri, V. Candela, F. Postacchini

Dipartimento di Scienze dell'Apparato Locomotore, Università di Roma "Sapienza" (Rome, IT)

**Introduction** The bone amount that should be removed during an acromioplasty has always been a challenge for the surgeon. Our aim was to verify the reliability of the existing data, to assess the differences between the two genders, to verify the existing correlation between scapular dimensions and acromial thickness and to investigate the relationship between acromial type and thickness.
**Materials and methods** We examined 500 dried scapulae, measuring three distances for each one referring to the scapular body and acromial thickness; they were also catalogued according to gender. Acromial shape was classified according to Bigliani's method. Chi square test was used to evaluate the association between variables; *t* test and ANOVA to evaluate the differences between groups. Pearson's correlation was also calculated.
**Results** The frequencies were: Type I 38.9 %, Type II 39.4 % and Type III 21.7 %, the mean of the acromial thickness was 0.85 cm; differences due to gender were stated. The correlation indexes showed a direct linear relationship between scapular dimensions and acromial thickness. While analyzing the acromial thickness, we found a statistical significant difference between hooked and flat acromion as well as between hooked and curved acromion.
**Discussion** The average value and the range of the acromial thickness proved to be wider than previously reported in literature. Because of the thicker acromion, male patients can have a more extended subacromial decompression, with a lower risk of fracture. The same reasoning applies to Type III acromia, which are averagely thicker (0.1 cm) and they also own a wider range of thickness with respect to the others Types. Acromial thickness proved to be correlated to the body habitus of the patient.
**Conclusions** While planning an acromioplasty, we should be aware that gender, specific scapular dimensions and acromial shape should be evaluated preoperatively since they influence the acromial thickness, in order to decide the amount of bone removal.

### Functional evaluation and MRI of shoulder rotator cuff: heritability estimates in monozygotic and dizygotic twin pairs

V. Arceri*[1], P. Albino[1], C. Fagnani[2], L. Nisticò[2], C. D'ippolito[2], S. Gumina[1]

[1]Dipartimento di Scienze dell'Apparato Locomotore, Università di Roma "Sapienza" (Rome, IT);
[2]Reparto di Epidemiologia Genetica, Centro Nazionale di Epidemiologia, Sorveglianza e Promozione della Salute, Istituto Superiore di Sanità (Rome, IT)

**Introduction** The aim of our study is to establish if (a) the shoulder functionality, (b) specific anatomic characteristics predisposing to tendons lesions, (c) rotator cuff tear are genetically determined parameters.
**Materials and methods** In the Italian Twin Registry, we identified 50 twin pairs of average age similar to that of patients with rotator cuff tear. We examined 31 twin pairs, 16 MZ (10 males; 6 females) mean aged 63y (range 53–72y) and 15 DZ (4 males; 8 females; 3 opposite sex) mean aged 63y (range 60–66y), without disease of the shoulder. All subjects underwent to: functional and subjective evaluation of the right shoulder (Constant Score; Simple Shoulder Test) and MRI. On the MRI images, we measured: acromio-humeral distance, angle of glenoid retroversion, area of the supraspinatus muscle; and we valued: degree of acromio-clavicular arthropathy, rotator cuff condition and Goutallier's stage.
**Results** Correlations of the three morphometric parameters were greater in MZ compared to DZ. The correlation of the acromio-humeral distance has been the highest (0.90 MZ; 0.24 DZ). Similar results have been obtained for the CS (MZ = 0.7; DZ = 0.5) and SST (MZ = 0.8; DZ = 0.6). From these correlations derived heritability estimates of 32 and 34 % for the CS and the SST, respectively. Higher values of heritability included: glenoid retroversion (31 %) and acromio-humeral distance (91 %). The correlation of the variable (degeneration/tear of the infraspinatus tendon) resulted greater in MZ (0.91) compared to DZ (0.44). Excluding age and sex, a short acromio-humeral distance was significantly associated to a higher risk of degeneration/tear of the supraspinatus tendon (OR = 1.77, 95 % CI: 1.13–2.77, $p = 0.01$) and of the infraspinatus tendon (OR = 1.73, 95 % CI: 0.93–3.23, $p = 0.08$).
**Discussion** Our data suggest that genetic factors play a preponderant role in determining the interindividual variability of the considered morphometric parameters.
**Conclusions** This study is the first that uses the "twin design". Our results suggest that the variability for the acromion-humeral distance and the degeneration/tear of the posterior-superior rotator cuff in each twin pair depends more from genetic factors than from environmental.

### Shoulder resurfacing in osteoarthritis: surgical technique and mid-term results

G. Merolla[1], P. Bianchi[2], R. Rossi*[2], G. Porcellini[1]

[1]U.O Chirurgia Spalla e Gomito, Azienda AUSL Rimini (Cattolica, IT);
[2]Dipartimento di Scienze Ortopediche, Traumatologiche, Riabilitative e Plastico-Ricostruttive, Seconda Università di Napoli (Naples, IT)

**Introduction** Shoulder arthroplasty remains the treatment of choice for gleno-humeral osteoarthritis. The modularity of the modern prosthetic systems allow us to choose the implant best suited to the case to be treated. In this study we assessed the mid-term outcomes after shoulder resurfacing in osteoarthritis.
**Materials and methods** From March 2005 to August 2011 we performed 984 shoulder arthroplasty, using the resurfacing in 60 cases (6 %) (mean age: 49 years; M/F: 36/24; dominant shoulder: 43 cases). Operations were performed under general anaesthesia with the patients in the beach chair position using a delto-pectoral approach and a lesser tubeorosity osteotomy. The mean follow-up was 44 months. Pre and postoperative radiographs included AP and axillary views. We implanted a humeral prosthesis in all cases and when we found a severe glenoid arthrosis, we performed: biologic resurfacing with omologue meniscus (n = 22), eccentric reaming and bloody microperforations (n = 10), biologic patch (Restore; DePuy Orthopaedics) (n = 4). All patients were assessed with the Constant-Murley (CS) score (mean + SD). Statistical significance was set at 5 % using the Student's t test for paired data.
**Results** CS increased from 48.2 (SD:6.4) to 68.6 (SD:4.5) ($p < 0.05$). X-rays showed a significant narrowing of joint space (5.92 mm postoperative vs. 1.65 mm at 37 months) in the cases treated with meniscus interposition while glenoid erosion was depicted in other 7 cases. Four unsatisfied patients with painful and stiff shoulder (follow-up: 31 months; CS: 52.6 + 1.4) underwent to meniscus removal and glenoid debridement (3 cases) and conversion in total arthroplasty (1 case).
**Discussion** Resurfacing arthroplasty is an effective device in young patients with aadvanced gleno-humeral arthropathy but biologic resurfacing appear not adequate to treat glenoid arthritis where the prosthetic replacement remain the best available option.
**Conclusions** Resurfacing arthroplasty is a good alternative to the total shoulder arthroplasty when the humeral bone stock is ensured but long-term FU studies are required to evaluate efficacy and survivorship.

### Is rotator cuff tear size influenced by high blood pressure?

S. Gumina[1], V. Arceri[1], S. Carbone*[1], P. Albino[1], D. Passaretti[1], V. Campagna[2], C. Fagnani[3], F. Postacchini[1]

[1]Dipartimento di Scienze dell'Apparato Locomotore, Università di Roma "Sapienza" (Rome, IT);
[2]Ospedale Militare Celio (Rome, IT);
[3]Istituto Superiore di Sanità (Rome, IT)

**Introduction** The aim of our study was to establish if hypertension may increase the risk of occurrence of rotator cuff tear and influence the size.
**Materials and methods** We studied 408 consecutive patients (228 M, 180 F) mean aged 59 year old (range 47–68) who underwent arthroscopic treatment for a rotator cuff tear. Dimension of the tear was determined in each patient at the time of operative intervention. For analysis, patients were placed in one of the two groups based on presence or absence of hypertension. We applied a logistic regression model to investigate if hypertension affects the risk of occurrence of a tear. Then, we fitted a multinomial logistic regression model to explore the association between hypertension and each type of tear. Furthermore, we used the ANCOVA method to determine if duration of hypertension influences the severity of tear; finally, we compared mean duration of anti-hypertensive therapy across small, large and massive tear patients.
**Results** The hypertension was associated with a two-fold higher risk of tear occurrence (OR = 2.05, 95 %CI: 1.41–2.98). While no association was detected between hypertension and the probability of a small tear (OR = 0.63, 95 %CI: 0.33–1.19), hypertensive subjects were twice more likely to experience a large tear (OR = 2.09, 95 %CI: 1.39–3.16) and four times more likely to experience a massive tear (OR = 4.30, 95 %CI: 2.44–7.58) compared to normotensive subjects. Mean duration of anti-hypertensive therapy significantly increased from small tear (1.08 years) to large tear (3.20 years) to massive tear (6.34 years) patients (ANCOVA: $F(2,403) = 16.357$, $p < 0.0001$).
**Discussion** Our data were adjusted for age and gender in order to avoid the influence of the two parameters which are strictly involved in the natural progression of the tendons lesion and which may be responsible for the extension of tear dimension. We can assume that a wide insertion area results impaired because of microcirculation degeneration.
**Conclusions** The hypertension is more prevalent in patients with rotator cuff tears. We found a significantly higher mean duration of therapy for those having a more severe tear. Our data provide evidence that hypertension is a significant risk factor for the occurrence and severity of rotator cuff tears.

### Efficacy of marrow-stimulating technique in arthroscopic rotator cuff repair: a prospective randomized study

L. Deriu*[1], M.F. Saccomanno[1], F. Donati[1], S. Careri[1], A. Grasso[2], G. Milano[1], C. Fabbriciani[1]

[1]Università Cattolica del Sacro Cuore, Istituto di Clinica Ortopedica (Rome, IT);
[2]Casa di cura Villa Valeria (Rome, IT)

**Introduction** Arthroscopic rotator cuff repair has a very high rate of successful subjective and functional results. However, structural healing is between 50 and 80 %. Numerous surgical techniques have been described in order to improve tendon healing. The aim of the study is to evaluate the efficacy of marrow-stimulating technique by microfractures of the greater tuberosity during arthroscopic rotator cuff repair.

**Materials and methods** Eighty patients with a full-thickness rotator cuff tear underwent an arthroscopic single-row repair. Patients were divided in two groups of 40 cases each. In group 1, standard repair was performed; in group 2, microfractures of the greater tuberosity were performed to enhance tendon repair. Clinical outcome was assessed using the DASH score and normalized Constant score. Tendon integrity was assessed with MRI. The following baseline variables were considered as potential predictors of outcome: baseline scores; age; sex; arm dominance; type of work, timing of symptoms, location, shape, and retraction of rotator cuff tear; fatty infiltration of rotator cuff muscles; treatment of the biceps tendon; repair technique; and number of suture anchors. Multivariate analysis was performed to determine which predictors were independently associated with the outcome. Significance was set at $p < 0.05$.

**Results** Seven patients were lost at follow-up (2 in group 1 and 5 in group 2) for baseline characteristics. Mean DASH score was 28.6 points in group 1 and 23.3 points in group 2. Mean normalized Constant score was 92.7 points in group 1 and 94.5 points in group 2. Comparison between groups did not show significant differences. Tendon healing rate was 52.6 % in group 1 and 65.7 % in group 2, without significant difference between groups. Subgroup analysis for tear size showed that group 2 had significantly greater healing rate than group 1 for large tears ($p = 0.040$). Multivariate analysis showed that age, timing of symptoms, tear location, tendon retraction and fatty infiltration significantly and independently affected the outcomes.

**Discussion** Microfractures of the greater tuberosity should improve tendon healing after an arthroscopic rotator cuff repair.

**Conclusions** Microfractures of the greater tuberosity did not significantly influence clinical outcome, but we observed, with numbers available, that microfractures significantly improved healing rate of large or massive rotator cuff tears.

### *AT14—FOOT AND ANKLE*

### Functional performance of a total ankle replacement: a combined study by means of gait and video-fluoroscopic tridimensional analyses

F. Cenni*[1], A. Leardini[1], A. Ensini[2], C. Belvedere[1], L. Berti[1], D. Luciani[1], M. Romagnoli[2], S. Giannini[3]

[1]Laboratorio Analisi del Movimento, Istituto Ortopedico Rizzoli (Bologna, IT);
[2]Clinica Ortopedica e Traumatologica II, Istituto Ortopedico Rizzoli (Bologna, IT);
[3]Laboratorio Analisi del Movimento, Clinica Ortopedica Traumatologica II, Istituto Ortopedico Rizzoli (Bologna, IT)

**Introduction** Currently a number of different prosthesis designs for total ankle replacement (TAR) are available, though only one was developed to establish full compatibility between the shape of articulating surfaces and the geometry of the ligaments. This compatibility is provided by a fully conforming meniscal bearing free to move backwards and forwards on both metal components, respectively during ankle plantar- and dorsi- flexion. Careful tridimensional analyses should be carried out in patients after TAR to assess these claims. The aim of this study is to assess of the functional performances of this TAR design by traditional gait analysis (GA), together with very accurate motion tracking of the components by 3D fluoroscopic analysis (FA).

**Materials and methods** Twenty patients implanted with the BOX Ankle TAR (Finsbury Orthopaedics, Leatherhead-Surrey, UK) were analyzed at 12 months after surgery. GA was performed during stair climbing and descending using a 8-cameras motion system (Vicon Motion Systems, Oxford, UK), electromyography (ZeroWire, Aurion, Milan, Italy), and an established protocol for lower limb joint kinematics and kinetics. For the same patients and motor task, FA was performed on the same day using a standard fluoroscope (CAT Medical System, Italy) at 10 Hz and an established technique. Motion between the three components was calculated.

**Results** From GA, nearly physiological joint kinematic patterns were observed in both legs. Particularly, a range of flexion at the replaced ankle of 14° and 17° were observed during stair climbing and descending, respectively. A statistically significant difference between the operated and contralateral sides were found only in the hip and ankle range of flexion ($p < 0.05$). From FA, 2.1 and 3.1 mm of antero-posterior meniscal-to-tibial translation were coupled with flexion between the two metal components. A significant correlation was found between meniscal motion and range of flexion at the replaced joint (from GA).

**Discussion** A physiological functional performance at the whole lower limb and also at the replaced ankle seemed to be restored even for this demanding motor task. The meniscal motion and its coupling with flexion support the main original claims of this design. A more physiological joint function and similar to contralateral side, was found in replaced ankles with greater meniscal motion.

**Conclusions** The TAR patients analyzed show the behaviour claimed by the design, which allows to achieve satisfactory functional results during normal activities of daily living.

### A prospective study of non-animal stabilized very high molecular weight hyaluronic acid (NASHA) intra-articular injections in haemophilic patients with ankle arthropathy

C. Ruosi*[1], D. Rossi[1], S. Liccardo[1], F. Granata[1], A. Coppola[2], E. Marrone[2]

[1]Università degli Studi di Napoli “Federico II”, Dipartimento di Ortopedia e Traumatologia (Naples, IT);
[2]Centro di Riferimento Regionale per i Disordini della Coagulazione, Università degli Studi di Napoli “Federico II” (Naples, IT)

**Introduction** Haemophilic arthropathy is the consequence of recurrent joint bleeds in patients affected by severe haemophilia A or B or Von Willebrand Disease, leading to functional limitations and chronic pain, with negative impact on quality of life. Main target joints are knees, ankles and elbows. Few therapeutic options are available in ankle arthropathy. Temporary symptoms improvement was reported by repeated (3–5) intra-articular administrations of hyaluronic acid (HA). A non-animal stabilized very-high molecular weight HA formulation (NASHA) has been proven to achieve longer-term benefits in patients with knee and hip osteoarthritis.

**Materials and methods** Patients with ankle arthropathy undergoing a single NASHA (Durolane 0.5 ml, Smith&Nephew) injection were prospectively studied. Clinical assessment, including Gilbert Score (Pain, PGS, and Physical Examination, PEGS), pain scores (Visual Anologue scale, VAS and McGill Score, MGS) and generic quality of life evaluation (EQ5D), and magnetic resonance imaging (MRI) were performed at baseline (T0) and 1 (T1), 6 (T6) and 12 (T12) months after NASHA injection.

**Results** Fifteen ankles in 9 patients (7 severe, 2 moderate, age 21–45 years) were treated. A significant improvement of symptoms and functional outcome was reported at T1 vs. T0 (PGS 0.5–0.6 vs. 1.7–1.0, $p = 0.03$; PEGS 2.2–2.8 vs. 4.7–3.3, $p = 0.001$; VAS 18–19

vs. 71–26; MGS 0.8–0.7 vs. 2.8–1.1; $p < 0.001$; EQ5D 80–14 vs. 54–25, $p = 0.015$).

**Discussion** Data were not statistically different at T6 vs. T1, suggesting long-term benefits after NASHA injection. Only 4 patients presently reached T12. The positive experienced outcomes led to repeat treatment in these 4 patients, 13 months (mean) after first injection. No significant change in the European MRI Score was found throughout the study.

**Conclusions** NASHA viscosupplementation enabled long-lasting clinical benefits and improvement of quality of life in patients with ankle arthropathy. This single-injection treatment provides an approach reducing risks and costs of multiple intra-articular injections, well accepted by patients and with a favourable cost-utility ratio.

### *AT15—TRAUMATOLOGY*

### Recombinant human bone morphogenetic protein-7 for treatment of long bone non-union: an observational, retrospective, non-randomized study of 105 patients

M. Ronga*, F. Baldo, G. Zappalà, E. Paiusco, P. Cherubino

Dipartimento di Ortopedia e Traumatologa, Università dell'Insubria (Varese, IT)

**Introduction** Autogenous bone-grafting is frequently used in the treatment of fracture non-union. The donor-site morbidity and potentially limited supply of suitable autogenous bone are commonly recognized drawbacks. Recent studies advocated the benefit and safety of recombinant human bone morphogenetic protein-7 (rhBMP-7) in several anatomical sites. An observational, retrospective, non-randomized study on the use of BMP-7 in treating non-union in various sites has been carried out by the BMP-7 Italian Observational Study (BIOS) Group.

**Materials and methods** The clinical series included 105 patients. The tibia was the most common site involved (44 %). Atrophic/oligotrophic non-unions were noted in 85 % of cases. In 46 % of cases, the original injury was an open fracture. The average number of previous surgeries for non-union was 2.2 (range 0–13 surgeries). Autologous bone graft was used previously in 23.8 % of the cases. Radiographic and clinical assessments were carried out at progressive time intervals on the followings groups: BMP-7 + autograft (A: 50 patients) or BMP-7 (B: 49 patients) or BMP-7 with composite grafts in 6 cases. Treatment was defined as successful when there was a return to normal daily activity with no associated pain and X-ray evidence of new bone bridging the fracture site on more than one radiological view. The results from groups A and B were analysed focusing on the following variables: time of union, previous complications, the number of previous surgeries, previous autograft. The cases treated with a composite graft were excluded, because they were not comparable due to the complexity of the treatment.

**Results** The mean follow-up was 29.2 months. The last assessment showed an 88.8 % success rate with an average healing time of 7.9 months (2–21). At = 9 months there was overlapping between the unions recorded in the two groups (PA–PB = 1.5 %; CI 95 %: −0.149; 0.119) for all the variables considered.

**Discussion** The study shows the efficacy of BMP-7 in the treatment of non-unions even in cases where it was previously used autologous bone graft. The observational nature is a limitation of the present study. Prospective, randomized and controlled studies are necessary for evaluating the efficacy of BMP-7 to improve the performance of autografts and allografts.

**Conclusions** BMP-7 is a valid alternative to autologous bone graft for the management of complex cases of non-unions.

### Dislocation of the knee and popliteal artery trauma: our experience and literature review

A. Ascoli Marchetti*[1], V. Potenza[2], M. Battistini[1], M. Morelli[1], M. Barbante[1], M. Benedetti Valentini[2]

[1]Chirurgia Vascolare, Università di Roma "Tor Vergata" (Rome, IT); [2]Ortopedia e Traumatologia, Università di Roma "Tor Vergata" (Rome, IT)

**Introduction** The dislocation of a joint is among the more subtle causes of damage to arteries that can lead to limb ischemia, potentially leading to amputation. Early diagnosis of a vascular lesion is important for limb salvage.

**Materials and methods** Between 2001 and 2011 were treated for lower limb vascular injuries associated with trauma osteoarticular 71 patients. The popliteal artery was affected by trauma in 31 cases (43.7 %). These include dislocation of the knee was present in 6 cases (19.3 %). In these urgent patients underwent orthopaedic assessment examination and X-rays to confirm the dislocation and the exclusion of associated bone lesions. The angiography examination after the reduction of dislocation allows evaluating vascular lesions that can be mild illness or unknown, and highlight the presence of early occlusion of the popliteal artery with the possibility to evaluate the vascular upstream and downstream. In emergency regime is associated with the revascularization performed in most cases, with autograft reversed saphenous vein of the contralateral.

**Results** Revascularization was performed successfully in all cases. The postoperative course was complicated by wound dehiscence in 2 cases, treated and resolved with local dressings. During the follow up of 56 months (min 3–max 120) patency was 66 % (4/6), 1 patient had asymptomatic occlusion, 1 patient was lost after 2 years. In 3 cases (50 %) transient neurological sequelae were observed for over 3 months.

**Discussion** Even a minor trauma, such as an accidental fall, a dislocation may cause a vascular lesion with the risk of ischemia of the lower limb. It must quickly recognize this lesion for early treatment, and reduction of dislocation and then revascularization, in case of associated vascular lesions. In our experience, the revascularization was performed with posterior approach, after the reduction of dislocation or by median with approach to the above and below the knee. The neurological lesions associated are responsible for most of the morbidity at distance.

**Conclusions** In the presence of a dislocated knee, it should always consider the possibility of a lesion of the popliteal artery, even after minor trauma. The multidisciplinary approach and the proper timing of treatment of injuries justify the good results.

### Internal fixation of displaced proximal humeral fractures with Philos plate: complications and functional outcomes

R. Sisto*, R. Matteotti, A. Martinasso, A. Maderni, D. Testa, R. Panarese, B. Battiston

Traumatologia, CTO Torino (Turin, IT)

**Introduction** Controversy persists concerning the preferred treatment of displaced fractures of the proximal part of the humerus. The present study was undertaken to evaluate the results of open reduction and internal fixation of three and four-part fractures of the proximal part of the humerus and the functional limitations of patients.

**Materials and methods** We assessed the intermediate and long-term results for 161 consecutive patients with a three or four-part fracture of the proximal part of the humerus who underwent open reduction and internal fixation with screws and Philos plate. The Constant-Murley score and DASH score (Disability of the Arm, Shoulder and Hand) were used as evaluation tests.

**Results** After an average of 51-month of follow-up (min. 18; max. 96 month), 51 % of patients had an excellent result on the basis of the Constant-Murley score, 22 % of patients had a good score, 8 % of patients had a sufficient result, whereas 19 % patients had poor Results. The mean score at Constant test was 66 points (range from 20 to 100). The 18 % needs a second surgical treatment to remove plate to reduce pain. The results of DASH score are similar to the Constant-Murley score.

**Discussion** Open reduction and internal fixation with screws and Philos plate should be considered even for patients with complex fracture that are associated with a high risk for avascular necrosis of the humeral head. Also important is the correlation between age, sex and functional recovery: is better to be male and young than female and old (Constant-Murley 85 vs. 40).

**Conclusions** Open reduction and internal fixation with screws and Philos plate yields good functional results in most patients.

### Gamma nail osteosynthesis in pertrochanteric femoral fractures: problems and complications

P. La Floresta*, S. Miceli, A. Gennarelli, A. Cavero, M. Ialonardi, G. Mastroberardino, M. Rivellino

Ospedale A. Cardarelli (Campobasso, IT)

**Introduction** The population ageing has produced a substantial increment in fragility fractures. The estimated amount of pertrochanteric fractures in 2060 is 4.6 millions. Gamma nail is a versatile and safe osteosynthesis device, designed for the treatment of proximal pertrochanteric femoral fractures. The Stability of the fixation device and its minimally invasive implant procedure leads to rapid functional recovery and good long term results. However, perioperative complications are not uncommon.

**Materials and methods** The study is based on the data collected from the Department of Orthopaedics and Traumatology of Antonio Cardarelli Hospital in Campobasso, analysing the treatment of pertrochanteric fractures by gamma nail osteosynthesis, especially focusing on perioperative problems and osteosynthesis complications. From 2000 to 2011, the treatment of 1553 pertrochanteric femoral fractures has been recorded. 1,006 patients were treated by gamma nail, 382 by DHS screw plate, 165 by Ender nails. The average age of patients treated by gamma nail was 76 years (range 24–99). Female patients were 725 and males were 281. In most cases the fracture occurred after a simple fall.

**Results** Intra-operative problems and complications were reported in 102 procedures: distal locking screw incorrect placement, 63 cases; set screw temporary misplacement in soft tissues during the application, 9 cases; guide wire protrusion across the acetabulum, 5 cases; lag screw protrusion into soft tissues, 5 cases; lag screw protrusion into joint space, 1 case; guide wire rupture, 1 case; cutter rupture, 1 case; under nail's apex fracture, 1 case; necessity to perform an open reduction, 16 cases. Local postoperative complications were reported during the First 2 months after surgery in 28 cases: cut out, 6 cases; fracture below gamma nail, 7 cases; haematoma needing evacuation, 6 cases; infection, 9 cases. Fifty-three patients died during hospitalization. Postoperative transfusion was given in 95 % of cases. Spinal anaesthesia was performed in all patients but six.

**Discussion** Intramedullar gamma nail fixation allows to perform closed reduction surgery, with reduced blood loss, ensures good stability permitting early mobilization. Despite this, the perioperative complication rate is considerable and it is due to surgical mistakes, to the reduced mechanical strength of osteoporotic bone and to the overall bone fragility of patients affected from pertrochanteric fractures.

**Conclusions** Gamma nail is a safe osteosynthesis device but to get a good result and to prevent perioperative complications, performing an accurate surgical technique and strict respect of the surgical steps is essential.

### Tibial pilon fractures: a 5-year follow-up of a personal case study. Presentation of a surgical procedure

D. Pontoriero, L. D'andrea*, F. Ciccolo, F. Coglitore, L. Micciché, A. Foti, M.A. Rosa

Scuola di Specializzazione in Ortopedia e Traumatologia, Sezione di Ortopedia e Traumatologia, Dipartimento Specialità Chirurgiche, Università degli Studi di Messina (Messina, IT)

**Introduction** A tibial pilon fracture is a skeletal injury in the distal tibial meta-epiphysis which affects almost the totality of the tibial articular surface; a good restoration of the joint is related to a reconstruction of cartilage surface, associated with a sufficient ligament balance and with a good reconstruction of soft tissue. Possible complications include: infection, deformity, nonunion, ankylosis.

**Materials and methods** Our series examines 97 patients, 39 XX and 58 XY, treated between January 2002 and December 2009. Ruedy-Algower's classification was used by the Authors. The treatment was the EF in 47 cases, "a minimum" osteosynthesis in 19 cases, "a minimum" osteosynthesis and EF in 14 cases, ORIF in 17 cases, including 8 with technical MIPPO. Minimum follow-up is 1 year and maximum is 5 years. Pre-operative planning, was based on X-Rays, CT and CT-3D studies.

**Results** The goal of the treatment was: anatomical reduction, stable fixation, respect for vascularization and soft tissue, early mobilization. The results were analysed by a series of X-rays and evaluated by OvadiaBeals score.

**Discussion** In our experience, the EF is an alternative to internal fixation in selected cases: highly comminuted fractures, open fractures, closed fractures with severe soft tissue injury and polytrauma. There several possibilities of EF. The most used technique, in cases of severe articular joint comminution, is the transarticular assembly through "ligament-taxis", which provides good reductions. In some dynamic systems of EF it is possible to unlock the ankle joint and allow early mobilization of the ankle, keeping however the "pontage". After a comparison of the major publications in the related area, it is difficult to give a conclusive judgement on the relationship between the classification, the treatment methods and the results.

**Conclusions** We believe that the treatment of tibial pilon fractures needs a multidisciplinary approach. Our experience has shown the versatility of the EF, which thanks to its characteristics, does not cause the patient any discomfort and does not hamper any subsequent plastic surgery intervention. Probably the tibial-foot "bridge with ligament-taxis" is the most effective system of EF. After the stage of initial consolidation of the fracture, we believe that it is desirable to allow a modification of the system, for a natural ankle mobilization: the conversion of the unit with the adoption of a hybrid periarticular form or a T terminal form responds to this necessity. External fixation is compatible with internal ostesynthesis.

### The use of the demineralized bone matrix (DBM), platelet rich fibrin (PRF) and bone marrow concentrated (BMC) increases healing in patients with non-union

D.M. Donati*, L. Cevolani, T. Frisoni, M. Di Liddo, B. Spazzoli, B. Dozza, E. Lucarelli, S. Giannini

Istituto Ortopedico Rizzoli (Bologna, IT)

**Introduction** The non-union looks radiographically as a fracture callus not very evident or absent 6 months after osteosynthesis. The incidence varies from 5 to 10 % of fractures of long bones. Patients undergo a long period of immobilization and this fact causes the increase of the social cost of the disease. Our technique aims to the reduction of the period of immobilization and as a consequence the management costs of the disease.

**Materials and methods** Our technique includes the infiltration of the nonunion focus with platelet rich fibrin (PRF), bone marrow concentrated (BMC) and demineralized bone matrix (DBM). Outpatients and radiographic checks were carried out 3, 6 and 12 months after surgery, and then once a year.

**Results** From November 2008 to March 2010 we treated 14 patients (average age 35 years, range 18–53). The sites affected are tibia (n = 8), femur (n = 5) and radius (n = 1); two patients had an open fracture. The ostesynthesis was performed with intramedullary nails (n = 3), external fixators (n = 7) and plate (n = 4). All the patients had pain and could bear partial load. We performed our treatment after an average period of 14 months (range 5–38) from the fracture. The average follow up was 12 months (range 3–30). After 3 months from surgery, seven patients bore full load, did not feel pain and X-rays showed an increase of osteogenesis which permitted the removal of the external fixator in 3 patients. Two cases failed; in one of them the patient underwent another infiltration and in the other one we replaced the fixation.

**Discussion** There are many therapeutic strategies in case of non-unions and they include the replacement or dynamization of fixation, the osteosynthesis with or without a splint versus bone, the external fixation and the use of adjuvant (electrical stimulation, magnetic fields, bone grafting, use of BMP's). These procedures require that the patient reduce physical daily activity. The technique we suggest promotes faster healing, helps the patient to be able to bear load soon and thus it contributes to the reduction of the management costs of the disease.

**Conclusions** The application of demineralized bone matrix, platelet rich fibrin and autologous bone marrow concentrate lets a reduction of healing times in patients with non-union. It also gives the possibility not to wait for long after ostesynthesis so as to be able to perform the surgery that is minimally invasive and can be performed through the One Day Surgery Programme. These facts contribute to the reduction of the management costs of the disease.

### *AT16—SPORTS TRAUMATOLOGY*

### Double-bundle anterior cruciate ligament revision surgery (RACL) using fresh-frozen Achilles-tendon allograft

S. Zaffagnini, G.M. Marcheggiani Muccioli, M. Nitri*, B. Tommaso, A. Grassi, C. Musiani, T. Roberti Di Sarsina, F. Iacono, M. Marcacci

Istituto Ortopedico Rizzoli (Bologna, IT)

**Introduction** The purpose of this study was to evaluate a novel double-bundle RACL technique using an Achilles-tendon allograft.

**Materials and methods** Between 2002 and 2008, 30 patients underwent ACL revision surgery in our department by means of a fresh- frozen Achilles tendon non-anatomical double-bundle arthroscopic technique, with soft-tissue fixation by staples. The Achilles tendon was splitted to have a two bundle graft: one was passed Over-The-Top to reproduce the AM bundle and one inside the femoral tunnel to reproduce the PL bundle. Only one tibial tunnel was created. The mean patient age at surgery was 28.1 ± 7.5 (range 18–46) years; the mean follow-up was 5.0 ± 1.5 (range 3–10) years. Clinical evaluation was performed with Tegner Activity scale, EQ-5D Score, Knee Injury and Osteoarthritis Outcome Score (KOOS), International Knee Documentation Commitee (IKDC) evaluation form. Objective laxity measures were determined using a KT-1000 arthrometer. Magnetic Resonance Imaging (MRI) evaluation was used to assess the signal intensity of the graft at follow up.

**Results** The median Tegner score improved from 3 (range 3–4) to 6 (range 4–7) ($p < 0.0001$). Moreover all other mean clinical scores improved significantly at follow-up: KOOS score (from 65.8 ± 9.3 to 82.5 ± 10.9; $p < 0.0001$); EQ5D score (from 0.34 ± 0.16 to 0.77 ± 0.23; $p < 0.0001$) and subjective IKDC (from 49.2 ± 8.0 to 75.8 ± 16.8; $p < 0.0001$). The objective IKDC score improved from 2B, 10C, 18D to 6A, 18B, 4C, 2D. KT-1000 manual maximum test side-to-side mean difference at follow-up was 3.0 ± 2.2 mm. The 80 % of patients returned to the same Tegner level prior to ACL primary reconstruction at a mean of 10.5 ± 1.5 months after revision surgery. MRI evaluation revealed a good graft signal quality in 25 patients at follow-up.

**Discussion** The method proposed is simpler than other published techniques for RACL and double-bundle graft combines the advantages of tendon allograft with the security of cortical fixation. Clinical results after RACL are slightly inferior compared with those after primary ACL reconstruction, but they are comparable to other RACL techniques published in the literature.

**Conclusions** The presented technique combines the Achilles tendon allograft advantages to the possibility to perform an intrarticular double-bundle ACL reconstruction. At 5-year mean follow-up a good restoration of laxity and function was recorded in 80 % of patients.

### ORAL COMMUNICATIONS

### *C58—BIOMATERIALS 1*

### Meniscal regeneration by a new polyurethane scaffold: a 2-year minimum follow-up study

S. Zaffagnini, E. Kon, G.M. Marcheggiani Muccioli*, G. Filardo, B. Di Matteo, A. Di Martino, M. Busacca, A. Scarale, M. Marcacci

Istituto Ortopedico Rizzoli (Bologna, IT)

**Introduction** Regenerative approaches to prevent/delay knee osteoarthritis after a previous partial meniscectomy have been advocated to improve the reparative processes of joint tissues, and good results have already been reported in the literature at long-term [1]. Recently, a new polyurethane scaffold (Actifit™, Orteq Ltd, UK) has been introduced in clinical practice claiming better material properties to resist the high knee forces and therefore a better chondroprotection [2].

**Materials and methods** We performed a prospective clinical evaluation of 18 patients (11 males, 7 females, mean age 45 years) affected by a massive loss of meniscal substance either medial or lateral (13 and 5, respectively), associated with intra-articular or global knee pain and/or swelling, and treated with Actifit™ implantation. Nine patients also underwent associated procedures involving cartilage treatment or osteotomies. Patients were evaluated clinically and with MRI up to 2 years of follow-up.

**Results** Evaluation of the patients who underwent Actifit implantation showed good results at short-term follow-up, both from a clinical and imaging point of view. One patient had a reinjury playing competitive soccer after the 12 months evaluation and was excluded from the subsequent analysis. IKDC subjective score improved from 47.3 ± 17.5 to 72.9 ± 13.9 ($p < 0.0005$) at 1 year and 74,6 ± 15,3 ($p < 0.0005$) at 2 years of follow-up, but an even higher improvement (77.7 ± 15.7, $p < 0.0005$) was documented when analyzing patients without associated treatment of chondral lesions or osteotomy.

**Discussion** Short term results after Actifit implantation are promising and similar to first generation meniscal collagen scaffolds [3]. The surgical technique was simpler compared to these previous generation devices and this is probably due to its better material properties.

**Conclusions** Reported results documented a significant clinical improvement treating partial meniscal lesions with scaffold implantation, but long-term randomized studies are needed to confirm if the better structural properties will lead also to better long-term clinical outcome and joint protection.

**References**

1. Zaffagnini et al. (2011) Am J Sports Med 39(5):977–985
2. Verdonk et al. (2011) Am J Sports Med 39(4):774–782
3. Rodkey et al. (1999) CORR 367(Suppl):S281–S292

### Acetabular cups in trabecular titanium: short-term follow-up

A. Bistolfi*[1], F. Rosso[2], D. Deledda[2], L. Ravera[2], F. Lagalla[1], F. Galetto[1], G. Massazza[3], M. Crova[3]

[1]AO CTO M. Adelaide (Turin, IT);
[2]Università degli Studi (Turin, IT);
[3]AO CTO M. Adelaide, Università degli Studi (Turin, IT)

**Introduction** In recent years, the aim of the orthopaedic research is to develop new materials which may guarantee both clinical and radiographic good results and osseo-integration. Among these, the materials with porous structure achieved an important role. The purpose of this study is to analyze the clinical and radiographic results of a series of innovative cups type Trabecolar Delta-Titanium (TT).

**Materials and methods** From December 2007 to November 2010 a total of 83 Delta TT (Lima-corporate®) acetabular cups were implanted in 75 patients (8 bilateral, 34 women, 41 men). Average age was 66.6 years (range 38–86). Different cups were implanted: from 46+ mm to 60 mm diameter. Harris Hip Score was used for clinical evaluation and the standard radiographs were used to identify signs of loosening or mobilization. All the data were prospectively collected and statistically analyzed.

**Results** All the patients re-entered in the study, with an average follow-up of 24 months. In all cases HHS scores have been a statistically significant increase from the pre-operative to the last control ($p < 0.05$). 32 patients (35 hips) were evaluated at the last control. HSS-HS total score at the last follow-up was 92 (SD ± 15.4) points. At the end of follow-up, 3 out of 83 prostheses underwent implant's revision: 1 case for recurrent dislocations (at 17 months to first implant), 1 for aseptic loosening and 1 for septic one. Radiographic analysis was limited to 64 acetabular cups with a minimum follow-up of 12 months: all the cups were well positioned and in no case there were progressive radiolucent lines.

**Discussion** Delta TT cup ensured good clinical and radiographic results, as expected for a very short follow-up for hip prosthesis. The primary press-fit was immediate and complete both in compact or porothic bone. The mobilization was correlated to a over-reaming of the cup at the moment of the implant. However, since this material is very innovative and different from most commonly used materials, the early observation is recommend, especially to avoid catastrophic immediately failures. Under this point of view, the TT appeared as a safe material with good clinical performance and excellent immediate press-fit.

**Conclusions** The technology of Trabecolar-Titanium surface treatment is safe in the short term and could potentially be the future for the acetabular osseointegration.

### Quantitative analysis of polyethylene wear debris in relation to total hip arthroplasty failure

G. Gasparini, D. Riccelli*, G. Tedesco, F. Familiari, P. Pizzuto, O. Galasso

Ortopedia e Traumatologia, Università degli Studi "Magna Græcia" di Catanzaro (Catanzaro, IT)

**Introduction** The main cause of surgical revision of total hip arthroplasty is the aseptic loosening secondary to periprosthetic osteolysis. This phenomenon is mainly secondary to wear of polyethylene. Several factors such as the amount, size and shape of the debris together with their biological activity and the host response may affect the onset of osteolysis. The aim of this study was to evaluate the role of polyethylene debris in aseptic loosening of total hip arthroplasty.

**Materials and methods** We evaluated 35 patients which underwent revision surgery a mean 7 years (range 1–18) after first operation. For each patient a histological quantitative analysis was performed on five samples obtained during revision surgery: four samples were taken from the periprosthetic interface membrane (acetabulum, proximal, middle and distal third of femoral stem) and a fifth specimen was taken from the neo-synovial membrane of the joint. Removed cups were observed macroscopically and were divided into worn, not worn and broken, the latter were subgrouped according to the spontaneous or traumatic rupture of the implant. A correlation analysis between the number of particles of polyethylene and the pre and intra-operative variables of each patient was carried out.

**Results** The histological analysis showed a greater number of particles in the samples from the synovial membrane and the interface membrane of acetabulum and the proximal third of the femoral stem, with respect to the membrane surrounding the middle and distal femoral stem ($p < 0.05$). The number of particles of polyethylene derived from implants revised less than 7 years after surgery was significantly lower than in revision performed after 7 years ($p < 0.05$), with a direct correlation between the number of particles and the duration of the implant. Most of the cups were classified as worn and had the longest survival after the first surgery (average 9.5 years). The number of particles observed in the worn cups was greater than in the not-worn and traumatic broken cups ($p < 0.05$).

**Discussion** Our data confirm previous findings of Amstutz et al. and provide useful new information to physician daily performing surgery for total hip arthroplasty.

**Conclusions** This study may elicit future studies to investigate the pattern of wear of high crosslinking polyethylene wear.

### Artimplant's Artelon augmentation device in anterior cruciate ligament reconstruction

P. Gifuni*[1], R. De Pamphilis[1], C. Sala[1], C. Manzini[1]

A.O. di Desio e Vimercate, P.O.C. Carate Brianza-Giussano (Giussano, IT)

**Introduction** The Polyuretane Urea (PUUR), a new class of synthetic polymers, has proved to be well tolerated by the human body and may constitute a suitable material for Anterior Cruciate Reconstruction (ACL). This material, Artelon Tissue Reinforcement band (ARTIMPLANT AB Sweden) is designed for ingrowth and incorporation to support the ACL reconstruction; the biomaterial degrades through hydrolysis (50 % after 24 months in vivo) but at the same time the band maintains strength during repair and remodelling and after 4 years it still retains 50 % of its strength. The objective of the present study was to evaluate the feasibility of Artelon Augmentation Device in ACL reconstruction, with less autologous tissue sacrificing, in patients following an accelerated rehabilitation program.

**Materials and methods** 22 ACL reconstruction (arthoscopic unitunnel technique by one surgeon) with a double autologus semitendinosus (ST) tendon sutured on Artelon Band were performed from January 2011. Mean age was 23.2 years (range 17–39) and mean follow-Up (FU) 5.9 months (range 3–12). The patients were evaluated with standard knee score and functional strength test; knee X-rays, MR imaging and diagnostic scope "second look" was randomly performed at 3, 6 and 12 months after surgery.

**Results** Up to date, the clinical result, in the patients who completed the accelerated rehabilitation program, were good with full restoration of function and stability and no residual pain in the donor-site; no adverse effects such as bone tunnel widening, re-rupture or instability, cartilage damage, joint reaction or macroscopic inflammation (pathological synovitis).

**Discussion** Earlier augmentation devices had a rigid texture and thus not the mechanical properties of a normal ACL or a tendon graft; the rigidity directs most of the loading to the augmentation device, with failure due to stress-shielding or fatigue of the device as a consequence. Recently, in vitro and in vivo studies have shown that the novel PUUR band has a similar elasto-mechanical loading profile as a human ACL tested post-mortem; so this degradable and biocompatible augmentation device could be used in conjunction with the tendon graft to provide resistance to the mechanical load during the time for revascularization and reorganization of the tissue.

**Conclusions** In any case the early results are encouraging; then, in our opinion, this material (Artelon) shows interesting properties for future clinical ACL reconstruction.

### Biophysical stimulation with ultrasounds of human SAOS-2 osteoblasts seeded onto trabecular wood (Luffa aegyptiaca) scaffolds

L. Fassina*[1], L. Visai[2], G. Magenes[1], F. Benazzo[3]

[1]Dipartimento di Ingegneria Industriale e dell'Informazione, Università di Pavia (Pavia, IT);
[2]Dipartimento di Medicina Molecolare, Università di Pavia (Pavia, IT);
[3]Dipartimento di Scienze Clinico Chirurgiche, Diagnostiche e Pediatriche, Università di Pavia (Pavia, IT)

**Introduction** A recent study [1] showed the potential of trabecular wood as biomaterial scaffold for orthopaedic applications. In order to extend the use of trabecular wood to the field of bone tissue engineering, we developed a new culture technique involving ultrasounds [2] (Igea Clinical Biophysics, Carpi, Italy) and an in vitro culture of human SAOS-2 osteoblasts seeded onto trabecular wood (Luffa aegyptiaca) scaffolds.

**Materials and methods** In particular, the ultrasounds (average power, 149 mW; frequency, 1.5 MHz; duration = 5 min/day) were applied to the trabecular wood for 22 days after the seeding of 1 million of osteoblasts per scaffold.

**Results** DNA extraction showed that the cell number grew to 2.95 millions in the control without biophysical stimulation, whereas to 4.51 millions in the ultrasound stimulated wooden trabecular scaffolds ($p < 0.05$). The extraction of bone proteins (type-I collagen, decorin, type-III collagen, osteopontin, osteocalcin, osteonectin, fibronectin, and alkaline phosphatase) via a sample buffer and their quantitative evaluation via ELISA showed that the ultrasound stimulation significantly enhanced the secretion and the deposition of bone matrix ($p < 0.05$). The SEM images showed that, in the control cultures, the cells were few and not surrounded by bone matrix, whereas the biophysical stimulation elicited the development of osteoblasts/bone matrix clusters tending to cover the surface of the trabecular wood.

**Discussion** The results obtained have shown that the ultrasonic stimulation caused a greater cell proliferation and an increased deposition of bone extracellular matrix.

**Conclusions** All these observations reveal our "biomimetic strategy": onto the wooden biomaterial surface, the ultrasound stimulation caused an enhanced formation of a new biocompatible surface composed of bone cells and their extracellular matrix.

**References**

1. Aho AJ, Rekola J, Matinlinna J et al. (2007) Natural composite of wood as replacement material for osteochondral bone defects. J Biomed Mater Res B 83:64–71
2. Fassina L, Saino E, Sbarra MS et al. (2009) Ultrasonic and electromagnetic enhancement of a culture of human SAOS-2 osteoblasts seeded onto a titanium plasma-spray surface. Tissue Eng Part C 15:233–242

### Instrumental evaluation with 3 Teslan MRI with the paramegnetic MDC in long-term biphasic scaffold (Trufit) osteointegration

S. Giannotti*[1], M. Ghilardi[1], G. Dell'osso[1], G. Bugelli[1], N. Cazzella[1], F. Cartei[2], V. Zampa[2], G. Guido[1]

[1]Clinica Ortopedica, Università di Pisa (Pisa, IT);
[2]UO Radiodiagnostica, Università di Pisa (Pisa, IT)

**Introduction** The Trufit®, is a cylindrical scaffold consisting of a poligraft. The poligraft is a patented synthetic material, porous and resorbable, mainly composed of an amorphous copolymer (poly dl-lactic-co-glycolic acid and calcium sulphate). In Europe, these scaffolds are used especially in articular cartilage defects and osteochondral defects. The aim of this study was to assess in long-term clinical outcome and instrumental results with MRI the successful osteointegration.
**Materials and methods** In the Orthopaedic Clinic of Pisa Trufit was used for the treatment of one or more focal osteochondral lesions of the femoral condyles positive MRI with or without concomitant ligamentous or meniscal pathology. In our 46 patients (47 cases, medium age of 57.89 years with painful symptoms and a positive MRI for osteochondral lesion) were implanted with a total of 71 Trufit. Cases were followed up with a minimum follow up of 5 years: they are 5 patients mean age 63.4 years with a mean follow up of 5.95 years (minimum 5.25–6.25 years max). Patients were evaluated using the Lysholm Knee Scoring Scale and instrumentally, with MRI with and without contrast medium in order to assess the vascularization of the bone-scaffold and colonization of the implant. For this study we used a very high field MRI unit (GE Discovery 750) 3Tesla with dedicated coil for the study of the knee (surface knee coil). We performed SGPR dynamic sequences, LAVA and DWI with paramagnetic contrast agent.
**Results** The clinical evaluation by Lysholm Knee Scoring Scale provided a postoperative medium score of 94.6 (min. 84, max. 100). The imaging showed excellent graft integration in 87.5 % of cases (7 plug out of 8) while the data relating to the vascularization and the interface are still under evaluation.
**Discussion** The results obtained indicate a resolution of pain, clinical symptoms and slowing joint degeneration. The interpretation of imaging is comforting about the integration of the scaffold, but would need a longer follow up and histological confirmation in order to better assess the poor integration of the central part in some scaffolds over 5 years and in one case the lack of resorption.
**Conclusions** The surgical Trufit is a good surgical alternative, but we wonder whether it is necessary to pre-load scaffolds with stem cells, preferably already oriented toward the osteo/chondrocyte line.

### *C59—KNEE 7*

### Unicompartmental knee arthroplasty: all-polyethylene versus metal-backed in patient with medial osteoarthritis. A six-year follow-up study

V. Sessa[1], U. Celentano*[1], A. Ruggeri[2], M.A. Rosa[2]

[1]Ospedale Fatebenefratelli S. Giovanni Calibita, UOC Ortopedia e Traumatologia (Rome, IT);
[2]Scuola di Specializzazione in Ortopedia e Traumatologia, Sezione di Ortopedia e Traumatologia, Dipartimento delle Specialità Chirurgiche, Università degli Studi di Messina (Messina, IT)

**Introduction** Unicompartmental knee arthroplasty (UKA) represents the most appropriate surgical treatment for patients that have osteoarthritis just of medial or lateral compartment. With this kind of surgery we have shorter rehabilitation because of soft tissue are much more preserved. The purpose of this study is to evaluate the results of the same UKA with two different tibial components: all-polyethylene versus metal-backed.
**Materials and methods** Materials and Methods We considered patients just with medial osteoarthritis that had surgery from 2004 to 2009. Age, sex, BMI, comorbidity and the two different tibial implants (all-polyethylene and metal-backed) were evaluated. Patients were examined using two different international scale one "Clinician completed" KSS, one "Patient completed" KOSS.
**Results** Medium follow-up was 5 years, no patients were considered with a follow-up less than 3 years. 78 patients had medial UKA. Two patients had been excluded because they needed revision surgery; one with all-poly implant because of loosening of tibial component, the other one with metal-backed implant had medial tibial plateau fracture. Among the remaining 76 patients, 46 had a UKA with all-poly tibial component (22 male, 24 female), 30 had a UKA with metal-backed plateau (9 male, 21 female). The average age was 81 years (standard deviation 7.47). KSS average score was 94.5 (standard deviation 12.33) and KOSS average score was 84.9 (standard deviation 12.27) for patient with all-poly UKA. KSS average score was 93.8 (standard deviation 7.27) and KOSS average score was 89.2 (standard deviation 6.98) for patient with metal-backed UKA.
**Discussion** Patients selected for a UKA implant need absolutely to have some anatomical and biomechanical requirements as integrity of ACL and good conditions of femoro-patellar joint or at least mild osteoarthritis. Surgery has not to achieve any overcorrection of knee alignment to prevent any further damage of the compartment not affected. In patients selected properly we have good results in 94 % of cases.
**Conclusions** Our results are really good as we can find in National and International works. All-polyethylene UKA shows good results really similar to results of metal-backed UKA. Any further improvements of these results will be strictly related to any changes of surgical technique and a proper selection of patients.

### Efficacy of biophysical stimulation in improving functional recovery in autologus chondrocyte implantation: a prospective, randomized, controlled study

M. Collarile*[1], M. Cadossi[2], E. Chiarello[2], S. Setti[3], C. Zorzi[4]

[1]Equipe Chirurgia Articolare Sostitutiva e Chirurgia Ortopedica (C.A.S.C.O.), IRCCS Galeazzi (Milan, IT);
[2]Seconda Clinica Ortopedica e Traumatologica, Università di Bologna, Istituti Ortopedici Rizzoli (Bologna, IT);
[3]Clinical Biophysics, IGEA SpA (Carpi, Modena, IT);
[4]Divisione di Ortopedia e Traumatologia, Ospedale S. Cuore (Negrar, Verona, IT)

**Introduction** Cartilage lesions are the principal cause of disability in population. The high specialization of cartilage tissue is responsible for its poor healing potentiality making it difficult to replace or reproduce. Cartilage treatment often needs a combined approach: surgical and bio-engineeristic. Hyaluronic acid Membrane Autologus Chondrocyte Implantation (MACI) represents one of the possible treatments of chondral-osteochondral lesions. Nevertheless, this engineered tissue, embedded in an inflammatory environment, tends to change its chondral phenotype into a fibroblastic one with a consequential loss of mechanical characteristic. The efficacy showed by Biophysical Stimulation to promote anabolic chondrocyte activity and to reduce the inflammatory response suggests its application in tissue engineering techniques. The aim of our study is to evaluate the effects

Biophysical Stimulation on pain and articular function of the knee after MACI.

**Materials and methods** We performed a clinical trial on 24 patients treated with MACI for cartilage lesions of the knee grade III/IV. Patients were randomized into experimental (Biophysical Stimulation with I-ONE therapy 4 h/die for 60 days) or control group. No significant differences were present at baseline. Function and pain were assessed with the following international scores: Visual Analog Scale (VAS), SF-36, IKDC and EuroQoL at baseline, 1, 2 and 6 months after surgery.

**Results** We observed a significant reduction of VAS in the active group at two ($p = 0.049$) and six ($p = 0.019$) months after surgery. The mean variation trend of the SF-36 shows a worsening in the first month only in the control group ($p = 0.003$). At the second month the SF-36 shows a significant improvement only in the active group ($p = 0.007$). Only the active group obtained a significant improvement in the IKDC at 30 days ($p = 0.027$). At 2 and 6 months, both groups showed a significant improvement of the IKDC score. Only the active group showed a significant improvement in the EuroQoL at 2 months after surgery ($p = 0.019$), while at 6 months both groups showed a significant improvement compared to baseline. The patient percentage with good knee function was higher in the active group: 57 vs. 14 % at 2 months and 87 vs. 63 % at 6 months.

**Discussion** We believe that the anti-inflammatory effect of Biophysical Stimulation is crucial to obtain a faster recovery and better clinical outcome after surgery. The control of the articular microenvironment soon after MACI should promote better clinical results also at mid and long term.

**Conclusions** Biophysical Stimulation is effective in reducing the post-operative recovery after MACI.

### Treatment of grade I-III chondropathy of the knee with PRP: results at 1-year

F. Uboldi*[1], G. Vergottini[1], E. Usellini[2], W. Albisetti[3], M. Berruto[2]

[1](Milan, IT);
[2]Istituto Ortopedico G. Pini (Milan, IT);
[3]Università degli Studi di Milano (Milan, IT)

**Introduction** The use of platelet-rich plasma (PRP) in chondropathy is currently a very discussed topic. Recently several scientific studies regarding its use in traumatic injury or degenerative cartilage, have shown encouraging results regarding pain relief and resumption of physical activity.

**Materials and methods** Between late 2010 and February 2012 we submitted 37 patients, 20 males and 17 females, aged between 37 and 65 years old, with a chondropathy grade between I and III of the knee, according to the scale of Kellgreen-Lawrence, with pain and limitation in sport or work life, to a cycle of 3 intra-articular infiltration with PRP. The derivative is obtained with method DS-ACP (Arthrex). Patients were evaluated with follow-up repeated at 1, 3, 6 and 12 months with a visual analogue rating scale (VAS) and Lysholm Scale.

**Results** At a follow-up period of 9 months (range 6–12), 40 % reported improvement in symptoms (mean reduction of 3-point VAS), 40 % did not benefit and remained stable (0 VAS points of difference from preoperative) and the remaining 20 % noted a worsening of knee pain. Functional results at Lysholm scale follow this trend. The chronological evolution is characterized by a progressive improvement up to 9 months before stabilizing or indicates a worsening of symptoms. Two patients underwent surgery (1 coblation of cartilage and 1 implants of TKA) for the worsening of the symptomatology at 6-month follow-up. There were no side effects. The best results were obtained in the group of patients aged less than 50 years.

**Conclusions** Conclusions emerge from our results comparable to what is currently published in the literature. This method results in better results in younger patients (under 50 years) and degree of chondropathy lower (I and II). The results also tend to improve over the first 8 months and then to deteriorate. 70 % of patients receiving this treatment have prompted more than a year a new cycle of infiltration.

### Planning of the sagittal tibial cut in TKA

F.R. Ripani*, P. Sessa, A. Della Rocca, F. Gabriele, C. Curri, G. Cinotti

Clinica Ortopedica, Università La Sapienza (Rome, IT)

**Introduction** In TKA, the degree of inclination of the posterior tibial cut (tibial slope), may affect knee balancing and knee motion in flexion. The aim of the present study was to assess the variability of the tibial slope in healthy knees and to investigate whether the sagittal tibial slope (STS) changes when cartilage and menisci are included in the STS measurement.

**Materials and methods** We selected 80 MRI of patients complaining of mild to moderate knee pain with no evidence of postraumatic or degenerative changes in the joint. In each patient, after the sagittal longitudinal axis of the tibia was identified, the slope of both the medial and lateral tibial plateaus were measured (bone slope) on the bone profile. The measures were repeated including the upper portion of both anterior and posterior parts of the menisci and underling cartilage (meniscal slope).

**Results** The bone slope was on average 8° and 7.7° in the medial and lateral tibial plateaus, respectively. The meniscal slope averaged 4.1° and 3.3° in the medial and lateral sides, respectively, with a significant difference between the bone and meniscal slope. Such a difference was due to a greater thickness of the posterior portion of both menisci and cartilage compared with the anterior one. In the lateral side, a significant correlation was found between the degree of posterior bone slope and the difference between bone and meniscal slope. In particular, patients showing a marked bone slope (between 10° and 15°) had a thicker portion of the posterior part of meniscus than those showing a mild to moderate bone slope (0°–5°).

**Conclusions** The posterior slope of the tibial plateaus showed a wide range of variability, with a mean values of 8° in our series, when measurements were performed on the bone profile. However, when cartilage and menisci were included in STS measurements, the overall STS was significantly reduced compared with the bone slope. Such a reduction was found to be greater in patients showing a marked posterior bone slope and vice versa. When planning the sagittal tibial cut in TKA, it should be considered that the actual posterior slope of the tibial plateaus is 3°–4° lower than that measured preoperatively on plain films.

### *C60—PAINFUL PROSTHESES 7*

### Painful biarticular prosthesis: a new implant choice

N. Galvano*, G. Danna, G. Valenti, R. La China

Clinica Ortopedica, Università (Palermo, IT)

**Introduction** In our experience but also from the scientific literature, we observed the presence of painful biarticular prostheses in about

15 % of our patients. This led us to review not only the surgical technique but especially the use of new materials and new prosthesis.

**Materials and methods** From November 2007 to November 2009, we studied 156 fractures of femur prosthesis, among these biarticular implants were the first choice in patients over 76 years with femoral neck fracture. Patients were followed-up at 6 months and 1 year after clinical examination by X-rays and VAS exam.

**Results** Among 110 patients followed-up (70 %), 15 % of cases had a dysfunctional pain syndrome caused by acetabular overhead, without any alteration of the offset, shortening of the limb. Peri-calcifications were not present in the prosthetic group. To solve this problem, since January 2010 we adopted a dual mobility implants and 79 patients were checked in 6 months. There was no pain, alterations of mobility or off-set. We observed a complete mobility of femoral coxo.

**Discussion** The use of a double-prosthesis is a rational choice above all to reduce the burden for acetabular source of pain but also to try and give an excellent mobility close to the physiological one. In the light of this new experience, it should be reserved only to people over 85 years.

**Conclusions** The use of double hip prosthesis reduces mobility if not reset the multifactor pain from dentures, present in about 15 % of current systems, patients beyond 75 years. The reduction of acetabular overload and cotiloiditis present in the use of biarticular prostheses, improves recovery and the outcome of patient.

### Postero-stabilized total knee arthroplasty: our experience

P. Zedde*, A. Melis, A. Santandrea, P. Tranquilli Leali, A. Manunta

Clinica Ortopedica, Università di Sassari (Sassari, IT)

**Introduction** In the last 7 years at the Orthopaedics Department of the University of Sassari we chose to implant postero-stabilized total knee arthroplasty in the management of knee osteoarthritis.

**Materials and methods** Between January 2004 and December 2010 we implanted 384 NexGen LPS, with posterior stabilized design and excision of the posterior cruciate ligament. Patient mean aged 65 years, had a primary diagnosis of osteoarthritis (IV-V stage). Preoperative and postoperative evaluation was based on the Hospital for special surgery score. Clinical outcome and postoperative complication were documented in a period between 1 and 7 years after surgery. Instrumental evaluation was performed using anteroposterior and lateral radiography to asses tibio-femoral alignment and component positioning. We could not contact 23 of the 384 patients.

**Results** Using the Hospital for special surgery score, 26.8 percent of the patients had excellent result (97/361), 36 % (131/361) a good result, 8 % (29/361) a satisfactory result and only 1.3 % (5/361) poor results. Pain during rest and pain when walking were absent respectively in 241 and 196 patients. A mean flexion of 105° was achieved and complete extension was observed in 240 patients. The poor outcome obtained in 5 patients was due to DVT (deep venous thrombosis) in 1 case, depressive syndrome and obesity in the remaining 4 cases.

**Discussion** Total knee arthroplasty success depends on the restoration of the proper axis of the lower limb, components design and its positioning, bone fixation, correct ligament balance and stability.

**Conclusions** Our experience shows that knee prosthetic surgery allows for a great result in recovery of ROM and pain relief in patients with severe varus or valgus deformity. A correct surgical indication and patient motivation are essential to achieve a good result.

### *C61—BIOMATERIALS 2*

### Resorbable materials in hallux valgus treatment

F. Barca*[1], A. Saracino[1], M. Venturini[2], A. Facchini[3], A. Bagno[2]

[1]Hesperia Hospital (Modena, IT);
[2]Department of Industrial Engineering, University of Padova (Padua, IT);
[3]Limacorporate (Villanova di San Daniele, Udine, IT)

**Introduction** The main feature of resorbable biomaterials for implantable devices is to avoid a second surgical intervention to patients: device removal is unnecessary since it will be degraded by the human body. Biodegradation will occur by progressive hydrolysis of biomaterial, which will be eventually excreted by usual metabolic routes. RSB Implants fixation system (Lima Corporate Trauma, San Marino) is made of P(L-DL)LA—a lactic acid co-polymer, in the ratio 70:30, with viscoelastic properties—to assure stability in a programmable period of time and a rate of biodegradation related to patient's healing process. The purpose of the present work is to evaluate the clinical experience obtained with resorbable fixation devices, such as double threaded screws, used at the Hesperia Hospital's Foot's Surgical Division, since 2004.

**Materials and methods** From January 2007 to January 2011, 329 surgical interventions have been performed using biodegradable double threaded screws. 48 patients (47 women and 1 man), affected by hallux valgus, have been selected and evaluated (30 with Normal severity, 17 Mild and 1 Moderate). Their age was between 22 and 72 years with an average of 53 years; all patients have been treated with osteotomies, settling the bony parts together with an RSB Implants double threaded screw (Lima Corporate Trauma, San Marino). Surgery for screw insertion included the lateral release of the first MF; after entering the medial part of great toe's MF, a capsular incision and an esosteotomy have been done; then, translational osteotomy and metatarsal head's retro-position have been realized, followed by the fixation of metatarsal head with the screw.

**Results** After surgery, patients with Normal severity reduced their intermetatarsal angle by 3,7° (S.D. 2.9); 4,9° (S.D. 3.1) was the reduction gained in patients with Mild severity and, in the only patient with Moderate hallux valgus, the correction was 8°. No patient had relapse, or compatibility problems with the resorbable material. From 6 up to 8 weeks after surgery, it was possible to wear normal shoes, going back to normal activities.

**Discussion** RSB Implants fixation devices exhibit several advantages: no surgery is needed for removal; no metallic ions are released through bone tissue; post-operative radiographic evaluation is easy; the stress-shielding effect is reduced.

**Conclusions** In conclusion, in the light of our clinical experience, the P(L-DL)LA co-polymer and the evaluated devices can be considered effective and safe.

### Do short stems decrease the "thight pain"? DXA study on bone-integration of two "stemless" hip joint implants

G. Logroscino*[1], V. Ciriello[1], A. Ziranu[1], E. Pagano[1], G. Magliocchetti Lombi[2], P. Piciocco[3], F. Santori[3], V. De Tullio[4], C. Albanese[4]

[1]Dipartimento di Scienze Ortopediche e Traumatologiche, Università Cattolica-Policlinico A. Gemelli (Rome, IT);
[2]Casa di Cura San Feliciano (Rome, IT);
[3]Dipartimento di Ortopedia, Ospedale San Pietro-Fatebenefratelli (Rome, IT);

[4]Dipartimento di Scienze Radiologiche, Università La Sapienza, Policlinico Umberto I (Rome, IT)

**Introduction** New "stemless" hip implants, prospect many advantages such as bone tissue and joint geometry preservation, as well as a reduction of the "thight pain" due to the absence of conflict between the interface of the implant and the femur. Mini invasivity is one more advantage. However few studies have been published on this argument. The purpose of this study, is to describe the stem-bone interaction, as studied by DXA, of two stemless hip implants.
**Materials and methods** Thirty-two stemless THA patients from at least one year, have been divided in two groups: A: Proxima-De Puy (n.19) and B: NANOS Smith&Nephews (n.12). All the patients were studied by X-rays and DXA. Five different areas (ROI) modified by the Gruen zone model, were taken into consideration in order to correctly evaluate the stemless implants (short stem implants).
**Results** At one year both the implants showed an increase of the BMD around the implant, preserving adequately the metaphyseal bone stock. The Nanos implant demonstrated a statistically important ($p < 0.05$) higher BMD in the areas 3 and 4. In the remaining areas (1, 2, 5) there were no significant differences of the BMD which was comparable to traditional long stems implants.
**Discussion** At one year the new stemless implants have demonstrated optimal bone integration. No signs of stress shielding or other signs of mobilization or osteolysis around the implant area were observed. Some differences of BMD between the two groups depend on the different implant design that can determine a different force distribution among the lateral and distal zones.
**Conclusions** This preliminary study demonstrated an excellent load transmission as well as an optimal bone integration of the new stemless implants. As a consequence, considering the bone tissue preservation, the reduction of thight pain and the mini invasivity, stemless implants can be considered a valuable alternative to traditional long stem implants.

### Treatment of avascular necrosis with stem cells under arthroscopic control

F. Cerza*, A. Pecora, A. Carcangiu, V. Schiavilla, I. Di Vavo

U.O.C Ortopedia, Ospedale P. Colombo (Velletri, Rome, IT)

**Introduction** Avascular necrosis is a serious complication, frequently due to a trauma. Among the most frequent localizations there are the femoral head and the Talus. We present three cases of avascular necorsis treated in our Orthopaedic Unit in the last year.
**Materials and methods** In all cases the diagnosis was confirmed with CT and MRI. We treated the lesions with bone marrow stem cells taken from the iliac crest and placed under arthroscopic control. The first case was a necrosis of femoral head in a 12 years old girl. The lesion was diagnosed 6 months after a sports injury resulting in a neck femur fracture, treated with screw fixation. In this case the treatment was consisted in the removal of screws fixation and insertion of bone marrow stem cells taken from the iliac crest, prepared with Marrowstim system and placed in the bone tunnels derived by screws removal in the femoral neck. The second case was a 40 years old man that had an osteonecrosis of the talus as a result of a ankle sprain that initially produced a trabecular bone contusion of talus. In this case the treatment consisted in the execution of the bone tunnel in the talus for the insertion of bone marrow stem cells. The third case was a 50 years old woman who has developed an atraumatic osteochondritis of the talus initially treated with OAT. After 5 years the patient had a recurrence of the chondral lesion and subchondral bone involvement. In this case the stem cells were placed into the chondral lesion directly by arthroscopy. A fully off load period of 90 days was observed by the patient with necrosis of femoral head. The two patients with necrosis of the talus and osteochondritis remained off load for 30 days. In the following 30 days they were granted a partial load. The follow-up examination was performed at 30, 60 and 120 days with radiographic control at 30 and 60 days and MRI at 90 days after treatment.
**Results** In all cases there was a healing of bone necrosis and of painful symptoms.
**Discussion** The arthroscopic control has allowed a better definition of cartilage damage and the presence of depression caused by necrosis of the subchondral bone. Moreover this makes it possible to better plan the rehabilitation program.
**Conclusions** The arthroscopic control allowed a better definition of cartilage damage. Moreover this makes it possible to better plan the rehabilitation program.

### Genetic effects of trabecular titanium on MG-63 cell line: an in vitro study

V. Sollazzo*[1], A. Palmieri[2], L. Massari[1], F. Carinci[3]

[1]Clinica Ortopedica Università di Ferrara (Ferrara, IT);
[2]Laboratorio Interdipartimentale di Chirurgia Maxillofacciale ed Ortopedia, Università di Ferrara (Ferrara, IT);
[3]Istituto di Chirurgia Maxillofacciale (Ferrara, IT)

**Introduction** Pure titanium and titanium alloys are materials widely used in orthopaedics because of their mechanical properties, chemical stability, and biocompatibility. Recently, a new highly porous titanium biomaterial named Trabecular Titanium (TT) has been developed.
**Materials and methods** In this in vitro study, we tested the genetic effects of TT (Lima LTO) on osteoblast-like cells (MG-63) using DNA microarrays technology: cDNA microarrays provides the ability to comparatively analyze mRNA expression of thousands of genes in parallel thus showing activated and repressed genes by the presence of the TT.
**Results** Several genes that were significantly up- or downregulated have been identified. Globally, it was demonstrated that TT stimulates osteoblasts proliferation and differentiation, and reduces apoptosis.
**Discussion** Therefore, all these effects can contribute to improve the osseointegration of this material.
**Conclusions** These results encourage the clinical application of Trabecular Titanium to prosthetic devices.

### *C62—KNEE 8*

### Can knee arthrodesis be considered suitable nowadays?

M.A. Rosa*[1], G. Maccauro[2], V. De Santis[2], S. Barreca[1], G. Orlando[1], C. Fabbriciani[2]

[1]Scuola di Specializzazione in Ortopedia e Traumatologia, Sezione di Ortopedia e Traumatologia, Dipartimento Specialità Chirurgiche, Università degli Studi di Messina (Messina, IT);
[2]Dipartimento Specialità Ortopediche e Traumatologiche, Università Cattolica del Sacro Cuore (Rome, IT)

**Introduction** The coming of arthroprosthetic implants has led to the disappearance of some surgical procedures typical of the past that were the cause of joint function loss, for example arthrodesis. Nevertheless, there are neoplastic and/or infectious cases that may take arthrodeses into consideration, since it is the only feasible treatment apart from amputation.
**Materials and methods** In the last 10 years, 35 knee arthrodeses have been carried out by the Authors, of which 15 concerning neoplastic diseases and 20 concerning infectious diseases secondary to primary prosthetic implants. Of the last 20, 12 were instrumented arthrodeses with intramedullary pins and antibiotic cement; 1 with A.E.F.; 3 with crossed Steinmann nails + A.E.F.; 1 with crossed Steinmann nails; 2 with Ilizarov E.F.; 1 with femoral-tibial intramedullary nail. In 15 cases of neoplasm of the lower limb 13 arthrodeses were carried out with long femoral-tibial nail, in 1 case of osteogenic sarcoma of the femoral distal metaphysis in childhood, after lengthening-reconstruction with an Ilizarov device, arthrodesis was performed with a long femoral-tibial nail and in 1 case of recurring chondrosarcoma of 2°/3° grade, after failure due to infection of a modular-oncological prosthesis, arthrodesis with non-articulate and silver coated prosthesis permitted the avoidance of an amputation.
**Results** The minimum follow-up is 6 months and the maximum 20 years. The acceptance of the surgical treatment has been assessed, on a special form, considering that if this operation is well done it lasts for ever.
**Discussion** The surgical treatment, in infectious and neoplastic diseases, must conform to the anatomic situation and nowadays, due to the increase in the number of these pathologies, it has reached an acknowledged importance and a remarkable technological peculiarity. The succession of prosthesis explants/re-implants, due to the increase in antibiotic resistant infections, causes an ever greater destruction of the periprosthetic skeletal tissue, so that amputation is a possibility that can be avoided only by arthrodesis, now considered an obsolete procedure, but the advantages of which are long lasting. The Authors after a thorough analysis of their survey have indicated different tecniques for both costs and the suitability for performing arthrodesis.
**Conclusions** The sacrifice of a joint is a defeat for the surgeon, but when a patient's entire limb or life are at stake, arthrodesis should be taken into consideration.

### Role of computer assisted surgery in knee prosthetic surgery

R. Iorio, D. Mazza, G. Bolle*, J. Conteduca, L. Valeo, F. Conteduca, A. Ferretti

Ospedale Sant'Andrea (Rome, IT)

**Introduction** The aim of this study is to evaluate the accuracy and reliability of VISIONAIRE (Smith & Nephew, Inc, Memphis, Tenn) Patient Matched Cutting jigs in both planes and for both components and to compare it with an extra-medullar tibial instrumentation, by analysing data as detected by intraoperative use of VectorVision knee navigation software from BrainLAB (Redwood City, Calif).
**Materials and methods** 12 patients entered the study. Preoperatively all patient underwent a full-length weight-bearing radiograph in A-P and a MRI according to the protocol. All patients were operated with cemented posterior stabilized prosthesis sacrificing cruciate ligament (Journey BCS, Smith & Nephew, Inc, Memphis, Tenn) by the same surgeon using the VISIONAIRE patient matched cutting jigs. During surgery, once the extra-medullar guides were placed and fixed on the tibia, the orientation on coronal and sagittal plane was checked by the navigator and then he was compared with the data obtained by measuring the orientation of VISIONAIRE Patient Matched Cutting tibial jigs. Then the orientation of the femoral cutting jigs was recorded. An unsatisfactory result was considered an error = 2° in both coronal and sagittal plane for tibial components as a possibible error of 4° could result in aggregate.
**Results** On the coronal plane the mean deviation of the EM tibial guides from the ideal alignment (0°) was 0.7° ± 0.39° while that of VISIONAIRE was 1.29° ± 1.55° ($p = 0.22$). On the sagittal plane the mean deviation of the EM tibial guides from 3° of posterior slope was -1.62° ± 1.78° while that of VISIONAIRE was +1.16° ± 4.29° ($p < 0.05°$). On the coronal plane the mean deviation of the femoral guide from the ideal alignment was 1.2° ± 0.6 and in the sagittal was 3.7° ± 2.
**Discussion** This preliminary study documented a only fair accuracy of the method with a consistent risk of error of more of 3° especially in the sagittal plane. We could speculate that the problem on the sagittal plane depends on the fact that the preoperative protocol does not include a lateral X-ray projection of the knee and only includes AP standing X-rays of the straight-leg and MRI.
**Conclusions** The standard instrumentation in the hands of experienced surgeon could lead to a better alignment of the prosthesis as compared with an MRI based Patient Matched Cutting Jigs.

### An atypical painful medial unicompartmental knee arthroplasty: non-traumatic femoral component breakage. A case report

A. Impagliazzo*[1], S. Pelle[2], A. Formica[3], A. Mattei[3]

[1]Casa di Cura Addominale EUR (Rome, IT);
[2]Università degli Studi di Roma "Sapienza" (Rome, IT);
[3]Azienda Ospedaliera San Giovanni Addolorata (Rome, IT)

**Introduction** Persistent knee pain after arthroplasty represents a clinical observation in almost 20 % of patients. Painful prosthesis represents an unpleasant cause of dissatisfaction for patient after surgery and a challenging item for physician in determining and solving causes; in some cases, early revision surgery is required within 5 years. Even thought aseptic looseening and periprosthetic infection are the main causes of implant dysfunction; depth investigations should be oriented as well to uncommon problems.
**Materials and methods** Evaluation of a painful unicompartmental knee arthroplasty (UKA) is characterized by difficulties due to the smaller experience with unicompartmental implants in relation to total-condylar ones, and to troubles in discerning pain origin from prosthesis or anatomical residual structures. The authors present a case of an atypical painful UK prosthesis in a 55-year-old man 3 years after an Allegretto unicompartmental implant for medial condyle osteonecrosis: a misdiagnosed breakage of femoral component in knee arthroplasty. The implant was totally replaced with another cemented Allegretto UKA after minimal recutting of bone surfaces. Samples of the periprosthetic tissue were taken in order to obtain histological and microbiological evaluations; no relevant data were obtained. Prosthesis components were submitted to mechanical studies and quality tests.
**Results** In examining failure causes, the authors underline the importance in patient selection for unicompartmental knee replacement (UKR) and in achieving a well-balanced and aligned knee in order to reduce stress on the components that could otherwise lead to fatigue fracture in relation to original extremely rare factory defects.
**Conclusions** To our knowledge, this is the first report of a non-traumatic breakage of the femoral component after modern unicompartmental medial knee implant with cylindrical parallel pegs and longitudinal ridge, as improved to strengthen initial design of UKA.

### *C63—PAINFUL PROSTHESES 8*

### Activity and sport after total knee arthroplasty

A. Bistolfi*[1], L. Drocco[1], E. La Marmora[[], G. Gays[2], M. Paonessa[1], F. Galetto[1], G. Massazza[3]

[1]AO CTO M Adelaide (Turin, IT);
[2]Università degli Studi (Turin, IT);
[3]Università degli Studi, AO CTO M. Adelaide (Turin, IT)

**Introduction** The return to high level of sport and an effective functional recovery are important factors for the patients after a knee replacement. Analyzing the different types of training, proper stretching and muscular strengthening programs it's useful to determine how to improve clinical outcomes and athletic ability, thus preventing accidents and protecting the prosthesis. The purpose of this study is to analyze the correct rehabilitation program based on the specific allowed activities indicated in sports studies.
**Materials and methods** The analysis of the literature data was developed in PubMed, Medline and the Cochrane Central Registry of Controlled Trials. Keywords references were: knee arthroplasty, replacement, prosthesis, sports activity, level, sports activity level, high-impact sports. The limits set for this review were: humans, time (10 years old).
**Results** The end of the study was March 2012. It was possible identify many protocols based on different functional demands of patients with total knee arthroplasty. The recommended sports activities for patients with total knee replacement are: cycling, swimming, ballroom dancing, archery, walking and golf. Some activities are activities allowed only to expert skill level: low-impact aerobics, cycling, bowling, canoeing, hiking, horseback riding, fencing, tai chi. Some high-impact sports are not recommended because they are very dangerous for people with knee replacement. Rehabilitation exercises and muscular specific strengthening allow a shorter recover.
**Discussion** The analysis of these articles evidenced the importance of a specific postoperative rehabilitation program, which must be immediate and intensive (from the use of continuous passive motion to physical therapy) in order to improve the return to sport, but also to decrease the complications and to prevent injuries. However, in the literature, there are not evidence based studies comparing the effectiveness of the different protocols after total knee arthroplasty. On the contrary, most studies are retrospective or with short follow-up.
**Conclusions** Sports is not forbidden to patients with total knee replacement, but patients must be awarded that not all sports are allowed. The prognosis and return to sport are influenced by a proper patient education about the risks of each activity and the teaching of a correct and constant rehabilitation. It is necessary to validate specific protocols for each sport, related to each patient needs and abilities.

### Differential diagnosis of painful prostheses: algorithm and cases

B. Pavolini*, L. Marzona, M. Aliani

UO di Ortopedia e Traumatologia, ASL1 Massa e Carrara (Massa e Carrara, IT)

**Introduction** Since 1999 Laskin has identified ten rules facing the problem of painful prostheses without defining a precise diagnostic path. Pain as a symptom of alarm in a hip replacement is a common but nonspecific signal that requires a clear path to define the source and the possible implications for the survival of the prosthesis. In the case of a cemented hip prosthesis, groin pain is present with a frequency of 20–25 % postoperatively, while in the uncemented prosthesis from 3 to 28 %.
**Materials and methods** The purpose of the authors is to present a diagnostic algorithm, illustrated by case studies. Starting from the analysis of the symptom "pain", it will direct the path of differential diagnosis in a clinical rationale.
**Results** Considering the algorithm of differential diagnosis of pain in home prosthetic, it can be excluded primarily the possible extrinsic causes correlated with neurological disorders, vasculopathic, tendinous, periarticular and others less frequent. The correct interpretation of pain (temporal, anatomical and functional correlation with surgery) associated with a dedicated physical examination (neuro-vascular symptoms, dysmetria, functional testing and ROM) allows you to address rationally then using the diagnostic investigation of first level as X-rays, ultrasound examination and serological markers of inflammation. The results of these tests can guide subsequent clinical investigations assuming then possible such as mobilization, sepsis, periprosthetic fracture, instability, dislocation, osteolysis, algodystrophy, allergies and prosthetic failure. Given the greater frequency of clinical mobilization, the scintigraphic investigations (with labelled leukocytes for possible infection or with technetium for aseptic loosening hypothesis) are the most widely used second-level examinations. The bacterial identification by culture of synovial fluid has an accuracy of 90 % and allows switching to the therapeutic phase if it has a positive trusted. The execution of a CT and/or an MRI help define morphological details that in the clinical pictures less frequent help the diagnostic definition. Examinations such as PET or allergy tests may be used in selected cases in which the diagnostic orientation is, despite previous, ill-defined.
**Discussion** The presence of many potential clinical and diagnostic investigations in cases of multiple painful prostheses requires the rational application of a diagnostic algorithm that effectively comes from the clinical situation to a clear diagnosis that has to always be critical with respect to the survival of the prosthesis.
**Conclusions** The correct interpretation of the symptoms of pain in specific clinical findings can rationally guide the diagnostic in case of painful prostheses.

### Failure of acetabular component in ceramic-on-polyethylene total hip arthroplasty: case report and review of the literature

O. Moreschini*, L. Marcellini, R. Cannata, C. Villani

Policlinico Umberto I, "Sapienza" Università di Roma (Rome, IT)

**Introduction** Catastrophic polyethylene failure is an uncommon complication of ceramic-on-polyethylene total hip arthroplasty (THA) because of the favourable tribological characteristics of these implants. We present a case of catastrophic failure of the acetabular component in a ceramic-polyethylene bearing THA and review of the literature.
**Materials and methods** A 58-year-old woman presented to our department with a 4-month history of increasing pain and squeaking from his left THA. On physical examination, we noted a severe and painful limitation in the range of motion of her left hip, and a 1.5 cm shortening of the limb. She had previously undergone a left THA approximately 9 years before our evaluation. She had not been followed-up for many years.
**Results** Plain radiographs of his left THA demonstrated an eccentric position of the ceramic femoral head within the acetabular cup, which was indicative of severe polyethylene wear. The femoral stem appeared well fixed with no evidence of periprosthetic osteolysis. During revision surgery, we observed the wear of the central part of the metal shell and the integrity of its margins. This allowed us to keep the metal shell and replace the polyethylene liner only. This choice was made because of the low bone stock and the excellent integration of the metal shell. The femoral component was not revised. The alumina ceramic femoral head was intact with dark metallic staining and superficial surface wear. Extensive metallic debris was evident macroscopically throughout the periprosthetic tissue and was confirmed histologically to be metallosis. Two years after surgery, the patient is pain-free, walks without ambulatory aids, and control plain radiography shows no implant mobilization.

**Discussion** Several studies have suggested that the wear of ceramic polyethylene bearings in THA is significantly less than metal polyethylene bearings and only 8 cases were reported in literature in which is observed protrusion of the femoral head through the metal shell. Many factors have been correlated with accelerated wear, including increased patient activity, thin polyethylene liner, polyethylene-sterilization in air, component malposition, ceramic material, and femoral head size.

**Conclusions** This case illustrates the catastrophic failure of the acetabular component after 9 years of ceramic-on-polyethylene THA. The absence of follow-up for almost a decade prevented the physicians from making an early diagnosis of the wear and a possible early revision procedure at a stage where the metallosis would have been less extensive. This suggests the importance of regular follow-up especially in patients with multiple risk factors for accelerated wear.

### *C64—NEOPLASTIC DISEASES AND INFECTIONS 5*

### Epithelioid hemangioma: a benign tumour with metastatic potential?

C. Errani*[1], F. Traina[1], S. Calderone[1], A. Bondi[1], M. De Fine[1], M. Nanni[1], A. Toscano[1], C. Antonescu[2], J. Healey[2], C. Faldini[1]

[1]Ortopedia Generale, Rizzoli-Sicilia (Bagheria, IT);
[2]Memorial Sloan Kettering Cancer Center (New York, USA)

**Introduction** The controversy surrounding epithelioid hemangioma (EH) diagnosis, particularly when arising in skeletal locations, stems not only from its overlapping features with other malignant vascular neoplasms, but also from its somewhat aggressive clinical characteristics, including multifocal presentation and occasional lymph node metastases. Specifically, the distinction from epithelioid hemangioendothelioma (EHE) has been very controversial. The recurrent t(1;3)(p36;q25) chromosomal translocation, resulting in WWTR1-CAMTA1 fusion, recently identified in EHE of various anatomic sites, but not in EH or other epithelioid vascular neoplasms, suggests distinct pathogeneses.

**Materials and methods** We investigated the clinicopathologic and radiologic characteristics of bone and soft tissue EHs in patients with available tissue for molecular testing. Twenty patients were selected after confirming the pathologic diagnosis and FISH analysis for the WWTR1 and/or CAMTA1 rearrangements. Most patients with EH of bone were treated by intralesional curettage, while patients with EH of soft tissue underwent excision with marginal or wide margins, with the exception of one case that was treated by biopsy only.

**Results** Four patients had multifocal presentation, including one with loco-regional lymph node metastases. However, none of the patients died of disease and only four patients developed a local recurrence.

**Discussion** Despite the fact that our series is relatively small, our findings confirm that EH does not behave aggressively and thus is a benign tumour. In fact, although most patients received conservative treatment, their long-term prognosis was excellent, and none of them died of disease. In the current study, we found that 4 cases of EH had a multifocal appearance. Although we cannot disprove a multicentric origin for EH, we favour the theory of metastatic spread of the tumour from bone and soft tissue, given the intimate relationship that vascular tumours typically have with non-neoplastic vessels. However, we do not believe that metastatic potential necessarily means malignancy. The possible existence of benign metastasis is further supported by the behaviour of giant cell tumours, another type of benign bone tumour that can metastasize without producing a fatal outcome. However, it seems that we are limited to our subjective interpretations and that we must wait for molecular analysis before a more definitive and objective answer becomes apparent.

**Conclusions** Our results, using molecular testing to support the pathologic diagnosis of EH, reinforce prior data that EH is a benign lesion, characterized by an indolent clinical course, with occasional multifocal presentation and rare metastatic potential to locoregional lymph nodes.

### Bone graft substitute with osteoinductive potential: use of silicate substituted calcium phosphate (0.8 %) granules in bone benign lesions. The experience of the I.N.T. "Fondazione G. Pascale" in collaboration with A.O.R.N. "Santobono Pausilipon Annunziata"

G. Federico*[3], M. Gallo[2], G. Colella[2], P. Di Giacomo[3], V. Siesto[3], P. Guida[3], F. Fazioli[2]

[1]A.O.R.N Santobono Pausilipon, I.N.T Fondazione "G. Pascale" (Naples, IT);
[2]I.N.T. Fondazione "G. Pascale" (Naples, IT);
[3]A.O.R.N. "Santobono Pausilipon Annunziata" (Naples, IT)

**Introduction** Bone graft substitutes are necessary to provide support, fill voids and enhance biologic repair of skeletal defects, especially in bone tumour lesions in children.

**Materials and methods** Our experience is a retrospective analysis of 36 benign bone lesions filled with a synthetic bone graft substitute composed by silicate substituted calcium phosphate (0.8 %) granules (2–5 mm) and micro-granules (1–2 mm) (s.s.c.p.) treated from November 2010 to January 2012 (FU: 10–24 months, mean age 14 months). This bone substitute presents osteoconductive and osteoinductive potential due to the high level of porosity (80 %) and to the presence of silicate ions (0.8 %). Those characteristics optimize bone biological mineralization. All patients were treated with aggressive or simple curettage and filled with s.s.c.p. The location of the lesions was: the proximal tibia (9), distal femur (8), proximal humerus (3), phalanx of the hand (3), tarsal astragalus (3), metacarpal (2), heel (2), proximal femur (1), distal radius (1), ulna (1), blade (1), tarsal cuboid (1), fibula (1). The histology of the lesions was as follows: Giant cell tumor (11), chondroblastoma (10), chondroma (8), low-grade chondrosarcoma (2), simple bone cyst (2), Pseudarthrosis of graft (2), aneurysmal bone cyst (1). Serial clinical and radiological controls were performed every 3 months. The parameters used for the evaluation of repair of osteolysis are: reducing and structuring of the lytic lesion, integrity of the cortical of the bone.

**Results** In all cases after the second month, compared to the identified parameters, we can appreciate a reduction of the volume of the lytic lesion and a resorption of the bone substitute. There were no cases of infection, or allergic reactions.

**Discussion** Compared to phosphoocalcic type HA bone substitutes and calcium carbonate ones, the s.s.c.p. showed to be a good product because it's biologically absorbed and well tolerated.

**Conclusions** The osteoinductive potential is the golden standard in bone graft and bone graft substitute. Our series shows that s.s.c.p. presents a strong osteoinductive action with many consequent advantages such an earlier return to the load and social activities. It's well tolerated and it can be used also in children.

### *C65—KNEE 9*

### Correlation between tibial component alignment and implant-bone fixation in unicompartmental knee arthroplasty: a radiographic and radiostereometric study

P. Barbadoro*[1], A. Ensini[1], F. Cenni[2], C. Belvedere[2], A. Leardini[2], S. Giannini[1]

[1]Clinica II, Istituto Ortopedico Rizzoli (Bologna, IT);
[2]Laboratorio Analisi del Movimento, Istituto Ortopedico Rizzoli (Bologna, IT)

**Introduction** Unicompartmental knee arthroplasty (UKA) has shown good clinical results but also higher revision rates than total knee arthroplasty (TKA). According to Whiteside, the three surgical keys to UKA success are: soft tissue integrity, implant component fixation and prosthesis component alignment. The correct position of the tibial component affects the good long-term results. Tibial component aseptic mobilization is one of the main revision causes of the UKA and can be accurately measured with RSA. Ryd demonstrated, with a sensitivity of 85 %, that progressive migration of the tibial component with a maximum total point motion (MTPM) increment of greater than 0.2 mm between the first and the second post-operative year, after TKA, was associated with an increased risk of later aseptic loosening.

**Materials and methods** 23 medial Optetrak® (Exactech, Gainesville-Florida-USA) UKAs with an all-polyethylene tibial component were implanted between 2006 and 2008. Intraoperatively four tantalum beads in tibial metaphysic and four in polyethylene were inserted. Posterior slope and varus-valgus alignment of the tibial component were measured at the post-operative X-rays. Translations, rotations and MTPM (translation of the marker that moves the most) of the tibial component were measured at 3, 6, 12, 24 months, by using a specific software (Model-based RSA 3.2-Medis specials-Leiden-Netherlands). At the same follow-ups clinical scores were assessed using the IKS system.

**Results** The means and standard deviations of MTPM 3, 6, 12 and 24 months were respectively: $0.4 \pm 0.1$ mm, $0.6 \pm 0.2$ mm, $0.6 \pm 0.3$ mm and $0.7 \pm 0.3$ mm. The maximum value of translation and rotation at 24 months did not exceed 0.5 mm and 3.5° respectively. Varus alignment of the tibial component was significantly correlated to anterior tibial sinking, varus rotation, anterior and medial translation and to MTPM. Only one knee was at risk of aseptic loosening. IKS -Knee and -Function scores at 2 years were 70 and 60 respectively.

**Discussion** Excellent clinical and RSA results were found at 2 years from surgery. Varus alignment of the tibial component greater 5° was correlated to UKA instability. A similar migration was found between these UKA and TKAs with a similar articular conformity. Good implant-to-bone fixation with a low risk of aseptic loosening can be obtained for conforming all-polyethylene unicompartmental knee arthroplasty.

**Conclusions** Good clinical results and long-term fixation can be obtained if there are correct surgical indications and great experience, using an accurate surgical technique. No more than 5° varus should be allowed during the tibial cut.

### Supervision of postoperative pain in patients operated of total knee re-placement: comparison between two different analgesic techniques

A. Russo*, E.C. Lo Giudice, F. Mendolia, E. De Rose

Ospedale Umberto 1 (Enna, IT)

**Introduction** Treatment of pain in the immediate postoperative period after a TKR is a debated matter. Purpose of this work is to assess the differences between two different types of anaesthesia and post-operative analgesia, in an attempt to analyze any possible difference in the physiotherapy treatments relating them to the result of an average follow-up of 3–12 months.

**Materials and methods** From January to December 2011, 61 patients have undergone a TKR (Balansys Mathys®). Inclusion criteria have been: varus deformity inferior to 15°, absence of attendant pathologies (diabetes, venous deficiency). All things considered 34 patients have been included in the study divided into two groups: A) Anaesthesia through epidural catheter and postoperative analgesia with a continuous infusion of Naropina 2–3 mg/ml by 100 ml elastomeric pump 4 ml/h connected to the epidural catheter. B) Subarachnoid anesthesia or narcosis and postoperative analgesia through administration of continuous intravenous infusion of morphine 20–30 mg/day. All patients have been evaluated in the following 6 days through the gathering of NRS, heart rate, blood pressure, respiratory rate and the possible appearance of side effects (itching, nausea, vomiting, hypotension). They have been evaluated through the KSS 3, 6 and 12 months after surgery.

**Results** NRS average value in patients of group A was of 4 (3–6), whereas for the group B it was 6 (4–8). The presence of side effects appeared more frequent in patients of group B. The KSS form showed, after 6 months, average values of 75 (68–82) for the group A and values of 70 (62–79) for the group B. After 12 months the values were 77 (66–85) for group A and 75 (63–80) for group B.

**Discussion** An effective supervision of pain may positively influence the level of general satisfaction of the patient and their articular recovery thus making the physiotherapist's task easier in the days immediately following the surgery. An important role is certainly determined by the effectiveness and by the methods for medications administration especially in the immediate postoperative period. The use of local anaesthetic released in the epidural space has been indicated as an effective and not very perilous method.

**Conclusions** In the light of the parameters obtained our study picks out that epidural anaesthesia compared to systemic analgesia reduces, for patients who have undergone a TKR surgery, postoperative pain, the incidence of side effects and makes TKR surgery easier. The good functional result in both groups shows however an advantage for patients who underwent epidural analgesia.

### Effectiveness of PRP in knee chondropathy: our experience

N. Galvano*, M.C. Agnello, M. Lauria, R. Adelfio, V. Triolo

Clinica Ortopedica Universitaria (Palermo, IT)

**Introduction** At our Orthopaedic Clinic we wanted to demonstrate the effectiveness of PRP in the articular cartilage of the knee.

**Materials and methods** We selected 20 patients with first-and second-degree detected by MRI, aged between 35 and 50 years including 10 men and 10 women. They were treated with N. 3 infiltration of PRP and evaluated at a distance using VAS and WOMAC scale and MRI with contrast medium performed at 12 months after treatment.
**Results** There was a marked improvement in symptoms and joint function, using as parameter of evaluation the VAS and WOMAC. There was an increase of cartilage thickness measured by MRI with contrast medium at time 0 and 12 months.
**Discussion** The only concern is related to the doubt of maintaining the thickness of the articular cartilage over time.
**Conclusions** The use of PRP actually determines an increase in thickness of the articular cartilage and a clear improvement of the painful symptoms.

### Preliminary experience with patient-specific instrumentation and preoperative navigation in total knee arthroplasty: remarks and instructions for use

M. Bruzzone*, R. Rossi, F. Rosso, F. Dettoni, A. Marmotti, D. Blonna, F. Castoldi

Clinica Universitaria, Ospedale Mauriziano Umberto I (Turin, IT)

**Introduction** Patient-specific instrumentation (PSI) is a relatively new method that has been developed to restore mechanical or kinematical axis and potentially reduce component malpositioning in total knee arthroplasty (TKA). It is based on the intra-operative use of disposable positioning guides (made by the manufacturer using a preoperative planning on a CT or MRI of the knee) to place pins or directly perform bone cuts. Theoretical advantages of this technique are shorter surgical time, less invasiveness especially on the femoral canal, better alignment of the components, less blood loss, reduction of necessary surgical instrumentation. The purpose of this article is to present the results of our preliminary experience with this technique and possibly confirm the hypothetical advantages of this technique.
**Materials and methods** In this case control study we compared clinical (blood loss, operation time, postoperative course) and radiographic (positioning and alignment) of 9 TKA's using a patient-specific templating alignment technique (PSI group) with results of a second control group operated on by conventional intramedullary alignment technique, match-paired for sex, age, BMI (Traditional group).
**Results** Mean alpha angle in the PSI group was 94.4° ± 1.7°, mean beta angle was 89° ± 1.3°, gamma angle was 3.6° ± 1.3°, delta angle 92.4° ± 2.4°. Same angles in the traditional group were respectively 89.6° ± 2.8°, 91.8° ± 1.1°, 3.8° ± 1.8°, 91.6° ± 3.4°. The only statistically significant difference between the two groups was the beta angle. For the PSI group, we compared the values of these angles on the preoperative planning on CTs and on postoperative radiographs: mean difference was 1.1° ± 1.3° for alpha angle and 1° ± 1.2° for beta angle; in one single case we report a difference >3° for the alpha angle. Blood loss and postoperative pain differences between the two groups were not statistically significant.
**Discussion** Use of PSI showed no different results if compared to traditional TKA. A study considering a longer follow up and a greater number of cases would be fundamental to better define advantages and drawbacks of this technique. However we believe that our experience could help surgeons who are beginning to use this technique.
**Conclusions** In our practice, the use of PSI gave us the opportunity to obtain an extremely precise surgical planning (especially for femoral rotation), reducing the possibility of intra-operative error and preserving bone stock. Nevertheless, surgeon's experience remains fundamental in checking and in case correct bone resections and in soft tissue balancing.

### Medial patellofemoral ligament ligamentoplasties in case of objective patellar instability

R. Philippot[1], M. Stamilla*[2], F. Farizon[1], G. Gresta[1]

[1](Saint Etienne, FR);
[2](Catania, IT)

**Introduction** In recent years, many authors describe techniques for the treatment of chronic patellar instability based on the repair of the patella medial foreground and in particular the Medial PatelloFemoral Ligament (MPFL).
**Materials and methods** The purpose of this study was to analyze clinical and scannograhic outcomes in a prospective series of 50 MPFL reconstruction. The inclusion criterion was chronic patellar instability. We included 50 patients; the mean age was 21 years (range 15–39). The analysis of preoperative and last follow-up was performed clinically by the IKDC and Kujala score, radiologically by measuring the Caton index, the LAURIN and MERCHANT angles and the patellar tilt quadriceps contracted and relaxed. The operative technique was standardized; it was a MPFL plasty with the gracilis based on a patellar fixation with 2 anchors and a femoral fixation with an interference screw into a tunnel located between the adductor tubercle and medial epicondyle. The graft was tensioned to 10 N at 30° of knee flexion. A bone distal gesture was performed in cases of preoperative TAGT higher than 20 mm, patella alta or genu valgum.
**Results** The mean follow-up was 26.91 months. Clinically, the average gross IKDC increased from 51 preoperatively to 72 at the last follow-up, the average real IKDC went from 38 to 62, the Kujala score increased from 48 to 82. Radiologically the tilt was quoted stage 2 as MALDAGUE classification in 39 cases against 16 cases postoperatively. On CT, the average TAGT decreased from 16.1 to 15.86 mm and the average patellar tilt went from 27.67 to 18.14 decontracted and 24–16.2 in contracted. We did not identify any recurrence of dislocation at the last follow-up.
**Discussion** With excellent clinical results at the last follow-up, no recurrence of instability and a single postoperative stiffness, our results confirm those previously reported in the literature.
**Conclusions** At medium term, our technique of MPFL plasty has demonstrated its efficacy in the treatment of objective patellar instability with a significant improvement of the clinical examination and especially the absence of recurrent dislocation, but also a significant patellar tilt correction.

### The fractures of the knee: need for an integrated approach

D. Lecce*, M. Feola, C. Rao, E. Gasbarra, N. Habib, V. Tempesta, U. Tarantino

Università degli Studi di Roma Tor Vergata (Rome, IT)

**Introduction** Every year in Italy there are about 16,000 hospitalizations for patients who experience fractures of the load knee, about 40 % of these affect people over 65. Analyzing female patients, 75 % of fractures of the distal femur, 40 % of the tibial plate fractures and 62 % of the patella occur in people over 65. This epidemiological distribution should let consider the knee as a site in which the aging of the bone and the progression of the osteoporotic disease can lead, even after a small trauma, to a macro and micro-structural failure as well as for the other typical fragility fractures, such as the femur, vertebrae, wrist, proximal humerus and ankle. The surgical treatment requires an understanding of how osteoporosis and other factors (comorbidity, drug therapies, damage of soft tissue, abnormal vascularization, bone gap) affect the characteristics of bone and the process of fracture healing.
**Materials and methods** Between January 2010 and December 2011 we operated 52 patients (28 men, 24 women) with fractures of the knee, among them 25 of the proximal tibia, 11 of the distal femur and 16 of the patella. The middle age was 52.1 years. A diagnostic protocol was applied to all patients over-65 (n = 19, 36.54 %) to assess risk factors, laboratory tests, bone densitometry and radiography of the dorsolumbar column.
**Results** The results of blood tests and instrumental allowed a diagnosis of osteoporosis in 11 patients (58 %) and 6 had at least one prevalent vertebral fracture. This has allowed treating patients properly.
**Discussion** Orthopaedics, in the management of fragility fractures, should consider the risk of possible complications such as delayed union, nonunion, vices of consolidation and mobilization of the implants. In all cases we used a surgical approach aimed to bone fragility, with minimally invasive techniques, coated screws and osteoconductive/osteoinductive substances. At discharge adequate antiosteoporotic therapy has been given to reduce the risk of new fractures and to improve the outcome surgery at the same time.
**Conclusions** The high incidence of fragility fractures in typical locations leads to a higher inclusion and consideration of these fractures in programs for the prevention of re-fracture. However it is necessary to think that less frequent fractures involving the knee must be evaluated with a diagnostic and therapeutic protocol aimed to reduce new fractures in other locations, but especially to reduce the risk of complications.

### *C66—HIP 7*

### Our experience with the monoblock tantalum cup: clinical and radiographic outcomes at medium- and long-term

M. Spiga*[2], C. Doria[2], A. Zachos[3], P. Tranquilli Leali[3]

[1]U.O.C. Ortopedia e Traumatologia (Oristano, IT);
[2]U.O.C. Ortopedia e Traumatologia, San Martino (Oristano, IT);
[3]Clinica Ortopedica, Università degli Studi di Sassari (Sassari, IT)

**Introduction** The osteolysis secondary to wear of the polyethylene coating is the most serious long-term complication in total hip arthroplasty. Numerous studies have shown that the fixing with screws, the modularity of the system and lack of "bone-ingrowth" are associated with osteolysis. This study reports our experience on the use of tantalum cup designed to solve these problems.
**Materials and methods** Between 2000 and 2011 were performed 386 total hip replacements using a cementless acetabular monoblock elliptical tantalum. The mean age was 67.5 years. The clinical evaluation pre-and post-surgery was performed by using the card Harrys (HHS), the WOMAC and SF-36. A radiographic study, observer blinded, independent, was conducted by the method of De Lee and Charnley.
**Results** The average in a recent follow-up was 91. All cups have appeared stable with bone growth. The gap occurred in 19 cups in the post-operative radiographs, and all have been filled. Radiolucencies at follow-up: 2 % zone 1, zone 2 1.6 %, and no wider than 1 mm, was not associated with any migration of the cup and no complication related to it.
**Discussion** The tantalum cup provides excellent primary stability, a good bone integration and the absence of symptoms at follow-up period of 8.3 years. No complications were reported in association with the use of tantalum.
**Conclusions** The elliptical shape of the cups creates a taproot of interference with the spherical acetabulum. Tantalum has a high coefficient of friction substantially cancellous bone than other implant materials. This property facilitates the direct apposition of bone in order to increase the initial implant stability.

### *C67—NEOPLASTIC DISEASES AND INFECTIONS 6*

### Custom made prosthesis of distal tibia and ankle after resection for bone tumours: report of two cases

N. Salducca*[1], V. Ippolito[2], U. Prencipe[1], L. Favale[1], C. Zoccali[1], G. Marolda[1], R. Biagini[1]

[1]Istituto Regina Elena (Rome, IT);
[2]Ospedale Civile (Brescia, IT)

**Introduction** Authors present two cases of patients treated at two different institutions with a custom made prosthesis of distal tibia and ankle after resection for bone tumours.
**Materials and methods** At the Civil Hospital of Brescia and IFO-Istituto Regina Elena of Rome two patients were treated with a Custom Made Prosthesis of Distal Tibia and Ankle after resection for bone tumors. Diagnosis was osteosarcoma (one case) and Ewing's sarcoma (one case). The prosthetic stem proximally was fixed in both cases by press-fit and distally in one case by press-fit and one by cementation. Functional analysis (according to the MSTS) was performed.
**Results** Functional results were excellent in both cases: we observed a good active movement without lameness when walking. The MSTS score was more than 80 %. We haven't observed local recurrences or metastases. In one case (os) we have observed a distal stem loosening (March 2012) because the patient went in jogging, 12 km per day, despite our recommendations. Currently the patient is scheduled for implant revision.
**Discussion** The impossibility to reconstruct the distal tibia and ankle in the past has led to considerable problems regarding the high number of infections and stiffness with impaired gait consequence of arthrodesis for limb salvage.
**Conclusions** Implantcast prosthesis of distal tibia is an excellent way to improve ankle function after resection for bone tumours and it is an important way to avoid rigidity and abnormal ambulation especially in young adult patients, but of course daily jogging is too much for this implant.

### Functional reconstruction of total femur with a megaprosthesis

D. Campanacci*, G. Caff, G. Beltrami, G. Scoccianti, P. Cuomo, R. Capanna

Ortopedia Oncologica e Chirurgia Ricostruttiva, Careggi, CTO (Florence, IT)

**Introduction** Modular prostheses of total femur are a valid alternative to obtain the control of the disease and to avoid radical treatment in patients with wide lesion. These implants, thanks to their good functional results, are actually used in selected patients with massive bone loss due to non oncologic condition as failures of systemic hip or knee prosthesis or trauma's sequel. Objective of this study was a retrospective evaluation of our cases of total femur reconstruction with megaprostheses aiming to evaluate morbidity and implant survival.

**Materials and methods** From 2001, 13 patients (5F, 8 M) with a massive femur bone loss for malignant tumour (11 cases) and for trauma's sequel (2 cases) were treated at our Institution using a modular prosthesis of total femur. In all the cases a MegasystemC® (Waldemar Link®, Hamburg, Germany) was implanted. In 1 patient a massive allograft of proximal tibia was associated to the modular prostheses.
**Results** At an average follow up of 30 months, 2 major complications were found: a wound dehiscence healed after a surgical debridment and an aseptic dislocation treated with a closed reduction. 1 local recurrence occurred leading to a necessary quarter amputation. The functional result was satisfactory (excellent or good according to MSTS score) in 72 % of 11 patients evaluated at the last follow up (2 patients died for the systemic spread of the tumour).
**Discussion** Due to the improvement of the diagnostic techniques and due to the coming of new medicament, modular prosthesis of total femur actually let to achieve more conservative treatments in selected patients with huge femoral lesions.
**Conclusions** Despite the high local recurrence rates present in literature, probably explained by the extension of the lesion, modular prosthesis of total femur represent a useful tool in local control of tumour ensuring a satisfactory functional result.

### Resection of hip and pelvis sarcoma after a previous GCT: clinical and reconstructive considerations

M. Gallo[1], G. Federico[2], G. Colella[3], F. Sadile[3], F. Fazioli*[1]

[1]I.N.T. "Fondazione Pascale" (Naples, IT);
[2]A.O.R.N Santobono Pausillipon (Naples, IT);
[3]A.O.U. Federico II (Naples, IT)

**Introduction** Let's now describe an interesting case, due to the natural history peculiarity and the surgery procedure adopted.
**Materials and methods** In 1985, the patient was operated on the greater trochanter of the left femur from GCT (Giant Cell Tumour). Twenty-five years from then, the patient seems to have grievous symptomatology on the hip with weakness of the lower left limb. The NMR of the pelvis emphasises the outcome of the previous bone grafting procedure and the non homogeneity of surface tissues adjacent to the pertrochanteric region. The accentuation of the grievous symptomatology with difficulty to ambulation leads to a necessary ago-biopsy of the left hip. The histology emphasises a highly suggestive description of high-grade osteosarcoma, secondary to the previous GCT. A NMR highlights a morphostructural alteration on the proximal third of the left femur, hit by greater destroying phenomenons of the neck and trochanteric region. In its interior, the injury presents necrotic haemorrhaged areas and it determines compression in the sciatic nerve. A further damage interests the omolateral acetabulum with the initial involvement of the acetabulum cavity. We proceed to the surgical intervention of extra-articular resection of the left hip and of the proximal femur through the prosthesis application.
**Results** The treatment shows both oncological and functional results. The ambulation occurs with slight lameness, even if showing an improvement.
**Discussion** Dating back to 1985, the appearance of a GCT required a local cure with curettage and bone grafting. Fifteen years later, in that location, a sarcoma arises. The hypothesis is a paraneoplastic sense transformation of a GCT. The disease affects both the proximal femur and the hip, forcing the necessity of sacrifice of the hemi pelvis and the proximal femur. The reconstruction has needed the implement of a prosthetic acetabular cup (PEDESTRAL®) and a modular prosthesis of femur (OSS BIOMET®).
**Conclusions** The peculiarity of this event represents the reason of this presentation, for both the natural transformation of a GCT in a malign sense and as regards the surgical procedure adopted.

### Neoangiogenesis is reduced in chronic tendinopathies of type 2 diabetic patients

M. Abate*[1], C. Schiavone[1], A. Pantalone[2], D. Vanni[2], B. Flacco[2], A. Rossetti[2], V. Salini[2]

[1]Dipartimento Ecografia Internistica, Università "G. d'Annunzio" (Chieti, IT);
[2]Clinica Ortopedica e Traumatologica, Università "G. d'Annunzio" (Chieti, IT)

**Introduction** In diabetes, the prevalence of tendon degeneration is increased. As neoangiogenesis is impaired in several diabetic complications, aim of this study was to evaluate the neovessels formation in tendinopathies.
**Materials and methods** Patients aged >55 years were selected, and divided in two groups: (a) type 2 diabetic patients, and (b) non diabetic subjects. In both groups, those with ultrasound features of tendinopathy were included, and the intratendinous vascularisation was estimated by means of Power Doppler.
**Results** Ultrasound features of tendinopathy were observed in 104 diabetic subjects and in 221 controls. Neovascularisation, with higher PD scores, was found more frequently in controls, while lower PD scores were prevalent in diabetic subjects.
**Discussion** Neovascularization inside tendons is less represented in diabetic subjects.
**Conclusions** These findings may have relevance on pain perception, because vessel proliferation is associated to nerves ingrowth, and there is a relationship between neoangiogenesis and pain in overuse tendinopathies.

### *C68—KNEE 10*

### Computerized tomographic assessment in the diagnosis of patello-femoral malalignment in adolescents

F. Falciglia, A. Poggiaroni*, M. Giordano, A.G. Aulisa, V. Guzzanti

Ospedale Pediatrico Bambino Gesù (Rome, IT)

**Introduction** Patellofemoral malalignment constitutes a diagnostic and therefore therapeutic problem that is not always easily solved in adolescents. In the study of the knee extensor mechanism, conventional axial radiographic views of the patella have some limits such as lack of a reference plane, the difficulty in controlling bone torsion and in dynamic examination. CT allows for cross-sectional views of the patella during static and dynamic phase with the knee at 15°–20° of flexion without distortion or overlapping and with a high sensitivity. In static phase, 3 type of malalligment can be evidenced: tilted, lateralized, lateralized and tilted patella. Each of these presents different clinical issues and instrumental data.
**Materials and methods** Twenty-seven adolescents, aged between 11 and 17 years, with anterior knee pain with or without patellar instability and a control group of twenty adolescents were studied by computerized tomography to detect patellofemoral malalignment. The examination was performed with and without quadriceps contraction with the knee flexed to 15 degrees. Congruence angle and patellar tilt angle were measured both in static and dynamic phase; sulcus angle, trochlear depth and the distance between the trochlear groove and anterior tibial tuberosity (TA-GT) were measured only in static phase.
**Results** The differences between the knees of the control group and those of symptomatic patients were statistically significant for all parameters measured in the CT. The investigation showed the well-known types of patellofemoral malalignment with quadriceps relaxed (tilted, lateralized, lateralized and tilted patella) and, in 52 % of cases, revealed changes in type and severity with quadriceps contraction (in 41 %, lateralization and tilting were markedly more evident; in 11 %, the type of malalignment changed).
**Discussion** Our results confirm the importance of CT also in adolescence and emphasize the dynamic examination increases the sensitivity of the method: in a certain percentage of cases, malalignment is detectable only with the comparison between the static and dynamic phase.
**Conclusions** The results demonstrate that, in addition to assessment with quadriceps relaxed, computerized tomography with quadriceps contraction is a useful adjunct to diagnose and define the type of malalignment in adolescents. Since the type of treatment is in relation to the characteristics of malalignment, the dynamic CT allows obtaining more useful data for the surgical indication.

### Scorpio prosthesis: clinical and radiological results to over 10 years

S. Giannotti*, M. Ghilardi, G. Dell'osso, L. Fabbri, N. Cazzella, E. Pini, G. Guido

Clinica Ortopedica, Università di Pisa (Pisa, IT)

**Introduction** The purpose of this study is to evaluate the clinical and radiological results of total knee arthroplasty (Scorpio Prosthesis) in patients with minimum follow-up of 10 years.
**Materials and methods** This retrospective study has recruited 94 patients (101 total knee replacement implants) with rheumatoid arthritis and osteoarthritis that underwent surgical intervention between 1998 and 2001 at our Clinic. 25 patients were lost during follow-up and 27 patients died. We re-evaluated a total of 42 patients with 49 implants. The medium follow-up is 136 months. The medium age is 79.68 years (minimum 67, maximum 90 years). We used a subjective evaluation as the WOMAC score and an objective evaluation as the KSS. Using the Knee Society total knee arthroplasty roentgenographic evaluation and scoring system we evaluated the radiolucent line of the implant.
**Results** The results with the subjective form were of 76/100 average, while with the objective assessment was 175/200. The radiolucent lines were on the femoral shield in 28 % of cases in zone 1, on tibial plateau in AP projection 35 % in medial zone 1, and tibial plateau in lateral view in 28 % of patients in anterior zone 1 and 2. There were 3 cases of reoperation, 2 of patellar prosthesis implantation and 1 of tibial plateau cementation.
**Discussion** The long-term results are good, it is still a discrepancy between clinical and radiographic parameters (assessed by the surgeon) and the degree of patient satisfaction, because the evaluation is always better for the surgeon than the patient. Of 42 patients 23 patients underwent surgery for total knee also the contralateral knee. We believe these patients were operated bilaterally an index of satisfaction of the surgeon independent of the clinical-radiographic parameters.
**Conclusions** The total knee arthroplasty is a well standardized method and is technically well executed with excellent results with long term follow-up.

### Unicompartmental knee arthroprosthesis: mid-term follow-up

R. Valentini*, S. Bernobi, P. Dordolin, G. Fancellu

Clinica Ortopedica e Traumatologica, Università degli Studi di Trieste (Trieste, IT)

**Introduction** The Literature is producing encouraging data about the implant of unicompartmental knee prosthesis though the uncertainty of long-term results is still deep-seated in many Surgeons. We wanted to compare the medium-term results of unicompartmental knee arthroplasty with tibial component of type "all poly" and type "metal back" from a clinical and functional point of view.
**Materials and methods** We prospectively evaluated 50 patients underwent implantation of unicompartmental knee at the Orthopedic Clinic of the University of Trieste. Patients were divided into two groups (A and B) formed by 25 patients homogeneous for sex and age. In the patients belonging to the group A was implanted Mitus (Link) unicompartemental prosthesis with tibial component in "all poly" feature, and the patients belonging to the Group B have been implanted an Allegretto prosthesis (Zimmer) with a tibial component with "metal back." The mean follow-up was 48 months. All patients were evaluated using the Knee Society Score.
**Results** The average KSS (objective and functional) preoperative found to be respectively for group A of 48 points and for the group B of 49 points and the post-operative KSS was found to be of 95 and 94 respectively for the group A and one B. The ROM medium has resulted with 125 degrees (range 85–140 degrees) of flexion–extension for group A and 130° (range 90°–145°) for group B.
**Discussion** We found no differences between implants in which the tibial component was made in polyethylene (but the thickness to be used must be greater than 6 mm) and those with the tibial component built in "metal-back" option. There were no revisions or loosening in our consecutive implants.
**Conclusions** To achieve positive results that remain such in the medium to long term it is important to the careful patient selection and accurate placement of components of the UKA, and the relative correction of the axes must be as physiological as possible.

### The crucial role of rehabilitation after total knee replacement in haemophilic patients

C. Ruosi*[1], M. D'anna[1], S. Liccardo[1], D. Rossi[1], M. Di Minno[2], A. Coppola[2]

[1]Dipartimento di Ortopedia e Traumatologia, Università degli Studi di Napoli "Federico II" (Naples, IT);
[2]Centro di Riferimento Regionale per i Disordini della Coagulazione, Università degli Studi di Napoli "Federico II" (Naples, IT)

**Introduction** Haemophilic arthropathy is the consequence of recurrent joint bleeds in patients with severe haemophilia A or B and von Willebrand Disease. Main target joints are knees, ankles and elbows. In patients with advanced knee arthropathy total knee replacement (TKR) may enable resolution of pain, function recovery and improvement of quality of life.
**Materials and methods** We evaluated medium/long-term outcome of TKR carried out between 2004 and 2008 in 10 patients (age range 47–58 years) with severe haemophilia A and advanced knee arthropathy with complete joint stiffness. All patients were proposed to follow a rehabilitation program starting with passive exercises for a week and specific daily physiokinesitherapy for 4 months. This program included deep breathing exercises, active ankle range of motion (ROM), lower-limb isometrics including quadriceps, hamstrings and gluteral sets, full and progressive isotonic and isometric knee and hip muscle strengthening. Hydrokinesitherapy was practiced from the second to the fourth post-operative month and every 6 months thereafter.
**Results** After knee surgery all patients achieved 90° knee exion minimum and full knee extension. At follow-up (range 3–7 years) clinical assessment showed that 3 out of 10 patients achieved a significant improvement of ROM with full knee extension and flexion >90°. These patients completed the post-operative rehabilitation program and continued to follow hydrokinesitherapy cycles.
**Discussion** Four patients showed full knee extension but flexion of maximum 80°. These patients were not fully compliant to the rehabilitation program. The remaining 3 patients did not maintain complete knee extension and their flexion was 40° in average. These patients were poorly adherent to the rehabilitation program since the early post-operative phase.
**Conclusions** In spite of the small study sample, our data remark the crucial role of an intensive and continuous rehabilitation program in determining functional outcome after TKR. Efforts should be devoted to improve adherence of patients to rehabilitation programs.

### ACL reconstruction with BPTB: optimization of the technique and results

M. Della Monaca*, M. Capone, A. Fascetti, D. D'Adamo, G. Lensi

Ospedale Versilia (Lido di Camaiore, IT)

**Introduction** ACL injuries are one of the most frequent lesions in Athletes. Surgical treatment aims to bring stability of the knee and resumption of the previous sporting activities. We describe our experience in ACL reconstruction with BPTB in a group of high demand athletes.
**Materials and methods** From September 2008 to September 2011 we selected and treated 78 patients: 54 male and 24 female with an average age of 25 years. All patients were assessed with an average follow-up of 26 months (12–36), using three evaluation sheets: Lysholm, Tegner and IKDC.
**Results** There were no complications such as patella fractures, infections, TVP, Cyclope syndrome and joint limitation. All patients resumed previous sporting activities.
**Discussion** During the surgical procedure, we paid particular attention to the anatomical placement of femoral tunnel, using the medial accessory portal and flexible drills. For femoral fixation we used the cortical suspension device Zip-Loop.
**Conclusions** BPTB is an excellent solution for ACL reconstruction in H–D athletes with respect of surgical anatomical reconstruction.

### Initial experience with Vanguard® total knee arthroplasty

M. Surace*, L. Monestier, F. Buggé, L. Murena, P. Cherubino

Dipartimento di Biologia e Scienze della Vita (DBSV), Clinica Ortopedica e Traumatologica, Università degli Studi dell'Insubria (Varese, IT)

**Introduction** Biomet Vanguard® Complete Knee System is a total knee replacement system, introduced into the market in 2003: this system boasts a designed kneecap track or groove which allows for greater mobility and decreased loss of healthy bone during the replacement process. Clinical and radiographic results in Varese Orthopaedics Unit initial experience are reported.
**Materials and methods** Clinical and radiographic parameters are evaluated in 29 patients who underwent TKA (Vanguard® Complete Knee System- Biomet Inc.) for primary gonarthrosis during the last 2 years at Orthopaedics and Traumatology Unit of Ospedale di Circolo—Fondazione Macchi in Varese. ROM, type of anaesthesia, patellar pain, clinical and functional Knee Score (pre-operative), size of implant, ROM, blooding, patellar pain, VAS, clinical and functional Knee Score (post-operative) are analyzed. Radiographic follow-up is made at 6 and 12 months.
**Results** Clinical and Functional Knee Score respectively improve from 47 to 88 pts and from 51 to 88 pts in pre- and post-operative period ($p < 0.001$). Post-operatively no patellar pain, 2 pts VAS and 1215 cc blooding are reported. ROM doesn't significantly improve from pre- to post-operative. No relevant complications are reported. Due to the short follow-up, radiographic evaluation does not allow to demonstrate polyethylene liner wear.
**Conclusions** Vanguard® Knee Replacement significantly shows a clinical and functional improvement in patients affected by primary gonarthrosis. Moreover, post-operative pain decrease allows early mobilization and rehabilitation.

### *C69—HIP 8*

### The large diameter in hip replacement surgery: a clinical and radiological analysis of our experience 4-year after implantation

F. Muresu*[1], C. Doria[2], P. Tranquilli Leali[1]

[1]Clinica Ortopedica, Università degli Studi di Sassari (Sassari, IT); [2]U.O.C. Ortopedia e Traumatologia, San Martino-Oristano (Oristano, IT)

**Introduction** Currently the use of new metal–metal coupling joints, ceramic–ceramic and metal-crosslinked polyethylene has allowed to considerably reduce the wear of materials that become an integral part of the prosthetic components. In hip replacement surgery the heads of large diameter are used due to its advantages will primarily related to lower risk of dislocation and greater range of motion that results.
**Materials and methods** We conducted a review of case studies with a follow-up to 3 years of 84 patients in whom a hip prosthesis was implanted with a large diameter head. Patients were operated at the Orthopaedic Clinic of the University of Sassari in the period between 2007 and 2010. The average age of patients was 70 years. In all cases the arthroplasties were implanted with a cementless acetabular component press-fit fixation and meta-diaphyseal stems.
**Results** The analysis was conducted by standard X-rays that made it possible to appreciate the good integration of the prosthetic acetabular component and the femoral stem, was also carried out a careful clinical examination, which was followed by the administration of the questionnaire Harris Hip Score. We have found no cases of dislocation or loosening of the components. The follow-up was at 3, 6 and 12 months.
**Discussion** At six-month follow-up control patients reported no pain in the hip operated at rest, standing and walking on flat ground, mild pain going up and down stairs was present in 9 patients. The range of motion was evaluated through the analysis of hip flexion in a sagittal plane, the decline in total average was 104° (range 90–130). This result is significantly important, as in the control group of patients with a prosthesis made up of heads of 28 or 32 mm in diameter was less the result with a total average decrease of 85°. In 68 patients there was no physical limitation while in the remaining ambulation was limited due to disease in the contralateral hip or neurological or other problems.

**Conclusions** The clinical and radiological evaluations conducted on our series show that the heads of large diameter provide excellent results in the short and medium term, ensuring greater stability and increased range of motion ensuring a reduction of conflict between the stem and cup with a lower risk of dislocation over the heads of small diameter. Radiographic analysis showed no radiolucent lines or signs of bone resorption.

### Is there still a place for total hip resurfacing in 2012? Our experience with 3rd generation total hip resurfacing implants

A. Calistri*, L. Marcovici, M. Bove, L. Di Martino, C. Villani

Clinica Ortopedica, Roma La Sapienza (Rome, IT)

**Introduction** Total hip resurfacing continues its popularity as a valid treatment option in active young patients with osteoarthritis, which thanks to this procedure are able to return to their normal daily activities also in relation to the high functional demand. The data published so far in the literature show that patients undergoing on total hip resurfacing feature parameters of kinematics and kinetics (moments of abduction, extension times, and walking speed) that are very similar to the normal values of patients without osteoarthritis. Several issues with respect to the coating facilities were first identified, then resolved by the scientific progress and the improvement of the surgical techniques.

**Materials and methods** Since November 2010 we prospectively evaluated 40 Conserve Plus Hip Resurfacing implants (Wright Medical Tech). Patients were evaluated at pre- and post-operatory (1, 3, 6 months, 1 and 2 years) by a clinical examination, X-rays and hip specific and patient oriented questionnaires.

**Results** No implant needed a revision (total or partial) for any cause. All patients performed a dosage of Metal Ions at 2 years of follow up as an ordinary monitoring, this is indicated by the guidelines for the management of patients with type implant Metal–Metal. No problems of premature wear were found in these patients.

**Discussion** To date, survival rate at 10 of follow-up of total hip prosthesis in patients younger than sixty-five years, as reported by the Swedish Arthroplasty Registry, is 85 %. These unsatisfactory findings will require a commitment to find alternative solutions. Currently, the complications of hip resurfacing for any reason are rare in the selected patient (osteoarthritic- young—male) and the survival rate at more than 10 years is 96.5–99 % as reported in the National registers and in literature. According to the experience of our Institute, which passes through the use of 1° and 2° generation implants, we believe there is a definite role for this type of reconstructive surgery of the hip in the light of currently available literature.

**Conclusions** The need to comply with a strict surgical technique and new knowledge with respect to the coupling reactions to wear in metal–metal require careful patient selection and rigorous and systematic follow-up of patients. Prospective studies and a careful evaluation of long-term results are needed to clarify the role of this Implant system in the treatment of degenerative hip disease.

### Advantages of frontal access in minimally invasive hip replacement surgery: our experience

A. Cassaro*, A. Raitano, S. Sauna

ASP 2, P.O.Vittorio Emanuele (Gela, IT)

**Introduction** This study evaluates the clinical results obtained in the minimally invasive hip surgery performed through the anterior access.

**Materials and methods** The study includes 18 patients, 11 women and 7 men, who were treated with total hip arthroplasty, for the first time, using the technique of Innsbruck Nogler.

**Results** The clinical results obtained: early functional recovery, reduced blood loss, reduced functional muscle injury, were classified as excellent, good, fair and bad. They were also compared with the results of the anterior street. The results also included a patient satisfaction.

**Discussion** This study shows that, despite the time required to perform the surgery is slightly greater than that necessary for the transgluteal access, the use of the MIS Front has some advantages: the early excellent functional recovery, reduced blood loss (15 %), reduced muscle trauma (1/3 approx.).

**Conclusions** We suggest that long-term evaluation, at most of the learning curve, surely will improve these evaluations, demonstrating the many technical advantages and the primacy of satisfied patients.

### Porous titanium short stem vitae, first 300 implants

M. Lisanti*[1], C. Fioruzzi[2], U. Fusco[3], O. Mauro[4]

[1]Ortopedia e Traumatologia I (Pisa, IT);
[2]Ortopedia e Traumatologia dello Stabilimento Ospedaliero di Fiorenzuola (Piacenza, IT);
[3]Ortopedia e Traumatologia, Ospedale Galmarini Tradate (Busto Arsizio, IT);
[4]Ortopedia e Traumatologia, Ospedali Galliera (Genoa, IT)

**Introduction** Short stems have had in the last years a remarkable development and stem designs available are becoming more numerous. At the same time the wider series of patients and longer follow up are winning the initial misgivings of some orthopaedic surgeons about the rotational and vertical stability of this kind of stems.

**Materials and methods** The primary stability of a short stem is linked both to the design that to the surface. The topic of this paper is Stem Vitae Adler Ortho. This stem has a design that allows high primary stability and a titanium coating with a high initial grip due to porosity of the trabecular structure with interspace of about 700 micron (Ti-por®). In this report we show clinical and radiological results in the first 300 implants of this stem in 4 Italian orthopaedics units.

**Results** The first 300 implants of this stem have given satisfactory clinical and radiological results even though within a short follow-up (max. 22 months).

**Discussion** As demonstrated in the literature dimension and geometry of porous, materials have a fundamental rule in promoting bone ingrowth and biological stabilization of the implants to bone. The production technique of Electron Bean Melting [EBM] Adler-Ortho makes it possible to obtain optimum porosity at the peripheral level, which is ideal for the trabecular graft and consequently to ensure secondary osteointegration. The developed technology with Ti-por® titanium allows to cover Vitae Stem with an extremely rugged (3D pores 700 micron) envelop. With modularity, (27 different neck combinations) this stem allows a perfect restoration of the hip geometry.

**Conclusions** Currently in the market there are no other short stems with this coating (if we exclude the neck-preserving stem Parva Adler Ortho), which according to previous studies allow bone in-growth and consequently ensure secondary stem osteointegration. Vitae Stem was designed with the philosophy of minimal diaphy-seal invasion, metaphyseal load, cancellous bone saving and adaptability to the major part of the proximal metaphysic. Its small size allows safety and rapidity of use, less bleeding and absence of thigh pain.

### Clinical-radiographic evaluation on a short-term period of the first 150 patients operated with hip replacement by MiniMAX® anatomic stem throught anterior approach

R. Rossi*, G.M. Giudice, F. Mazzotti, M. Di Scioscio, M. Barchetti, P. Vasina

AUSL Ferrara (Argenta, IT)

**Introduction** The Authors analyse clinical and radiological results of 150 patients who were operated for cementless hip artroposthesis with MiniMAX® anatomic stem (Medacta International SA), between October 2007 and 2011, through anterior approach.

**Materials and methods** The prosthesis was chosen because it is designed following the modern concepts of shape and anchorage, which must be realised between femur and metallic implant to optimize "fit and fill". It is ananatomic stem prosthesis. The contemporary acetabular shell implant was done using the cup Versafitcup® CC (Medacta International SA), as press-fit, with ceramic insert. All surgeries were realised using AMIS® technique (Anterior Minimally Invasive Surgery, Medacta International SA). The anterior approach is the only technique that follows a path both intermuscular and internervous and therefore considerably reduces the risk of damaged periarticular structures. To facilitate the surgical phases and to make the surgery reproducible, we added an integrative extensor device to the standard operating table: the AMIS® Mobile Leg Positioner which allowed the hip to flex, extend, abduct, adduct and internally and externally rotate. The use of this device allowed the reduction of: (1) theatre assistants required throughout the procedure and (2) surgical phases reproducibility.

**Results** We found these relevant advantages: no muscles cut, significantly shorter rehabilitation, a small skin scar, decreased postoperative pain, less blood loss, reduced hospital stay, no dislocation, faster return to daily activities. In the short-term, the stem is radiographically well integrated to the bone with a good metaphyseal grip. No loosening has been highlighted.

**Discussion** The curved design of the stem on both surfaces, sagittal and frontal, facilitates the insertion through a small incision. Also dimensions, which are shorter respect to other prosthetic patterns, are suitable for a mininvasive approach. The clinical result of lack of thigh pain in all examined cases confirms the choice of a specific and unique tip design optimised to guide the prosthesis insertion into the femoral canal and to avoid distal interference.

**Conclusions** Very good efforts in short-term support a good synergy in choosing the implant of this anatomic stem with anterior approach.

### Use of new radiologic MRI and CT methods in the evaluation of hip painful prostheses

G. Ferretti*[1], A. Impagliazzo[2], S. Pelle[3], A. Formica[1]

[1]Azienda Ospedaliera San Giovanni Addolorata (Rome, IT);
[2]Casa di Cura Addominale EUR (Rome, IT);
[3]Università degli Studi di Roma "Sapienza" (Rome, IT)

**Introduction** Prosthetic designs and materials evolution, refinement of technique and precise instrumentation in modern hip surgery lead to excellent results in mid and long term period. However, unpleasant complications may occur with variable frequency related to fixation system (12–25 % for cemented prostheses, 3–28 % for uncemented); one of these the onset and persistence of pain. Several causes of pain could be recognized such as infection or aseptic loosening, implant breakage, periprosthetic fracture, neurological problems and more intra and extra-articular factors. Radiological investigations and haematological exams are often useful in identification of causes of failure in the great part of patients, whereas the unexplained painful arthroplasty still remains a challenge for the surgeon and a major cause of patient's dissatisfaction after surgery. These features caractherize a clinical defined set of painful prosthesis; not obvious and known clinical causes are not detected by depth investigations as well oriented to uncommon problems.

**Discussion** In the study of painful prosthesis, important contribution is currently offered by modern imaging techniques such as Computed Tomography and Magnetic Resonance, thanks to those who are the qualities of resolution and contrast specific for different tissues. The modernest techniques can overcome some limitations of currently used imaging. CT scan allows a better visualization of mineralized tissues and structures opacified by mc, MRI provides a multiplanar display with greater detail in resolving peri-skeletal tissues. MRI can also be carried out in the upright position allowing to acquire details in weight-bearing condition.

**Conclusions** The advanced CT and MRI can bring to radiologist and orthopaedic surgeons attention to morphological changes of recent diagnostic examination in patients with painful prostheses that could acquire dignity as proper disease entities and as unique cause of the disorder itself.

### RESIDENTS' ORAL COMMUNICATIONS

### *SPE01—RESIDENTS' ORAL COMMUNICATIONS*

### Single row versus double row arthroscopic rotator cuff repair; clinical and 3 Tesla MR Arthrography results: a retrospective study

C. Tudisco, S. Bisicchia*, E. Savarese, R. Fiori, D.A. Bartolucci, S. Masala

Università di Roma Tor Vergata (Rome, IT)

**Introduction** Two different groups of arthroscopic rotator cuff repair techniques are described in literature. Double row (DR) repair methods demonstrated many biomechanical advantages and higher rates of tendon-to-bone healing. However DR repair failed to demonstrate better clinical results than SR repair in clinical trials.
**Materials and methods** We retrospectively evaluated two groups of patients operated on for a medium size arthroscopic rotator cuff repair at a minimum 3 years follow-up. First group consisted of 20 patients operated with a SR technique, second group consisted of 20 patients operated with a DR technique. Clinical evaluation included Constant-Murley Score (CSM) and Simple Shoulder Test (SST). Radiological evaluation included 3 Tesla MR Arthrography (MRA).
**Results** The mean follow-up was 40 months in SR group and 38.9 months in DR group. The mean post-operative CMS was 70 in SR group and 68 in DR group. The mean SST score was 9.4 in SR group and 10.1 in DR group. The re-tear rate was 60 % in SR group and 25 % in DR group. Leakage of the contrast medium was observed in all patients.
**Discussion** MR imaging at 3 Tesla, especially with intra-articular contrast medium (MRA), demonstrated better diagnostic performance than 1.5 Tesla in the musculoskeletal setting, with a consequent improvement in bone and soft tissue detail. We think that leakage of the contrast medium is due to an incomplete tendon-to-bone sealing that is not a re-tear.
**Conclusions** To the best of our knowledge this is the first report on a 3 Tesla MRA in the evaluation of two different techniques of rotator cuff repair. DR repair provided a statistically significant lower re-tear rate, with longer surgical time and higher implantexpense, despite no difference in clinical outcomes. Leakage of contrast medium at suture anchor level could have important medico-legal implications.

### Mason II radial head fractures: conservative treatment versus reduction and internal fixation with absorbable pins

G. Gazzotti*, R. Loschi, M. Francia, C. Rovesta, F. Catani

Policlinico (Modena, IT)

**Introduction** In this study we compared conservative treatment and the use of bio-absorbable poly-p-dioxanone (PDS) pins, for Mason type II fractures of radial head.
**Materials and methods** In 18 patients a long arm plaster splint was performed for 25 days followed by above-elbow posterior splint application. Twenty-five patients were treated with open reduction and internal fixation using absorbable pins. The mean follow-up was 28 months in the conservative-group and 38 months in the pins-group. Functional outcomes were assessed with DASH questionnaire, MAYO score, elbow ROM and stability, strength compared to contralateral side and by reviewing the radiographs. A visual analog scale (VAS-scale) was used for evaluation of pain.
**Results** Union was achieved in all patients. There were not significant statistical differences between the two groups. There was an earlier mobilization of elbow in the pins-group (mean 15 days) than the conservative one (mean 25 days). In the conservative group, two patients reported an important flexion limitation.
**Discussion** Mason type II radial head fractures management is controversial. In the treatment of this radial head fractures, the radio-ulnar joint surface must be reconstructed to avoid earlier arthrosis, stiffness, pain and to maintain the useful motion of elbow.
**Conclusions** The use of absorbable pins allows an earlier elbow mobilization and a good quality of reduction and stability, therefore we suggest this technique for young patients. In the case of patient with poor functional performances and when the fracture involves the part of radial head that not articulate with sigmoid notch, the conservative treatment can be indicated.

### Surgical treatment choice in Stener's lesions: our experience

A. Bini, G. Pilato, S. Ferraro*, A. Fagetti, P. Cherubino

(Varese, IT)

**Introduction** Stener's lesions, frequently associated with physical activity, are diagnosed with difficulty, and often undiagnosed. Diagnostic evaluation and choice of treatment depend on clinical, radiographic, and ultrasound evaluations. The literature reports several types of treatments for Stener's lesions. The aim of the present study is to evaluate postoperative results in relation to the type of lesion and the type of surgical technique, comparing them to results reported in the literature.
**Materials and methods** Between 1998 and 2010, 40 patients affected by Stener's lesion were operated at our Institution. The lesion was diagnosed clinically and radiographically, and confirmed intra-operatively. In patients with an unclear preoperative diagnosis and lack of bony lesions on radiograph, an ultrasound was performed. According to the classification by Saetta, 3 patients presented with a type I lesion, 6 patients with a type II, 16 with a type III, and 15 with a type IV lesion. The latter patients were further classified according to criteria by Kozin and Bishop. Mean follow-up was 80.3 months. Postoperative evaluation was based on criteria by Saetta et al.
**Results** Postoperative evaluation reported excellent results in 32 patients, good results in 8, and no poor result. In all cases, both optimal range of motion and stability of the first metacarpophalangeal joint were observed. All patients returned to work and sports after mean 3 months from surgery.
**Discussion** The choice of the surgical technique was always based upon intra-operative anatomical and pathological evaluation of the lesion. Type I lesions were repaired with a suture anchor; type II lesions were repaired by primary suture of the ligament; type III lesions were treated with suture anchors; type IV lesions were treated with K wires. If the bony fragment was too small to use K wires, the fragment was removed and the ligament repaired with a pull-out technique or a suture anchor. A review of the literature, and the excellent results in our patients, support the validity of the above-mentioned therapeutic approaches.
**Conclusions** We believe that the surgical treatment of Stener's lesions should depend on the type of lesion, as determined intraoperatively.

**Revision of prosthetic hip: relationship between bone stock and quality of life in aseptic loosening of the femoral component**

C. Villani, D. Attala*, L. Di Giorgio, M. Gurzì, L. Sodano, I. Toulopakis

Università Sapienza (Rome, IT)

**Introduction** In this study we evaluated the patients operated on for aseptic loosening of hip prosthesis to analyze their clinical and radiological results at the medium and long term. Then, we focused on bone quality of the patients studied, to understand which importance has bone stock quality for a good clinical result.
**Materials and methods** We examined 34 patients who underwent revision of hip arthroplasty for aseptic loosening of the hip prosthesis stem. The sample was divided into 4 groups classified according to Paprosky scale applied to the radiographic preoperative evaluation of femoral bone stock. The evaluation of clinical status preoperatively and at the end of follow-up was be made as questionnaires SF-36, Harris Hip Score and WOMAC. Radiographic evaluation of the patient's bone quality was performed on the standard A-P view of pelvis and on axial view, preoperatively and at 1, 6, 12 and 18 months after operation.
**Results** Data obtained from the sample were placed in the postoperative complications arisen in relation to the state of the femoral bone-loss before the surgery time. The groups PI and PII second Paprosky showed a high level of satisfaction with an average value of reference for the postoperative rated as excellent, the absence of complications and good functional recovery at the end of follow-up. Conversely groups PIII and PIV have shown increased incidence of complications and difficulties with clinical rehabilitation and SF-36 scores and HHS lower than other classes.
**Discussion** From what seems to emerge from the study, bone quality at the time of surgery affect the therapeutic target. In fact all patients with good bone quality at the time of the review (groups PI and PII) showed a distinct advantage from surgical treatment. Conversely, the revision surgery has not had much effect in patients with high bone loss.
**Conclusions** A strategy in anticipation, resulting in preservation of bone stock, should be considered a good foundation for the success of a prosthetic reimplantation with better functional and radiographic out-come associated with greater satisfaction for the patient.

### *SPE03—RESIDENTS' ORAL COMMUNICATIONS*

**Synthetic versus bovine hydroxyapatite in hip revision surgery: preliminary results at 18 months**

D. Vanni*, A. Pantalone, S. Sogari, F. Salerni, A. Puzzo, E. Andreoli, V. Salini

Clinica Ortopedica e Traumatologica, Università "G. d'Annunzio" (Chieti, IT)

**Introduction** Difficulties present in hip revision surgery are often correlated with defects in the bone tissue, especially in the acetabulum. These osteolytic lesions can be associated with the loosening of the prosthesis. The regeneration of the bone tissue is therefore necessary to give adequate stability and grant bone stock with regards to future revisions.
**Materials and methods** The aim of this study was to analyze if they are significant differences between hydroxyapatite (HA) of synthetic origin (Regenoss) or bovine origin (Orthoss) in the treatment of bone defects in hip revision surgery based on their biological and mechanical characteristics. In this prospective, randomized clinical study patients identified for hip revision surgery with an acetabular defect type I or II according to Paprosky have been included into the study. Patients have been either treated with the synthetic HA (group A) or the bovine HA (group B). Both groups were homogenous with regards to age (60–80 years), sex and general clinical findings. Patients were evaluated pre- and post-operatively at 1, 3, 6 and 12 moths follow-up by the Harris Hip Score (HHS) and X-ray analysis plus a CT scan at 18 months. Statistical analysis was performed after Kaplan–Meier and $p < 0.05$ was considered statistically significant.
**Results** Improvement in HHS revealed no statistical significance between the two treatment groups. Better results, although not statistical significant, have been evaluated in the CT scans of group B showing better osteointegration of the bone graft.
**Discussion** Autologous bone is still the gold standard as bone graft substitute, but its availability is limited as well as the associated donor site morbidity remains a problem. The availability of a bone substitute with the same characteristics of autologous bone but without the afore mentioned limitations, could be a new gold standard.
**Conclusions** The principle endpoint of hip revision surgery is to achieve immediate stability of the implant. Synthetic bone substitutes do not achieve similar characteristics of autologous bone although availability is not limited. The availability of a bone substitute of non-human origin but with macro- and microscopic similar characteristics of autologous bone is an important alternative for treating bone defects in hip revision surgery.

**Treatment of shoulder instability in professional athletes: arthroscopy versus open surgery**

D. Vanni*, A. Pantalone, M. Menna, M. Passeri, M. Natale, A. Rossetti, V. Salini

Clinica Ortopedica e Traumatologica, Università "G. d'Annunzio" (Chieti, IT)

**Introduction** Shoulder instability treatment has seen many important changes over the last years, thanks to the surgical techniques improvement, especially of arthroscopy. Post-traumatic shoulder instability is a common disease, especially in sportsmen. If not diagnosed and inadequately or late treated, it may be responsible for an articular biomechanics alteration, with serious problems in short and long term. This is much more obvious for professionals athletes, because corporate and market needs force them to a premature return to sport. The purpose of this study is to demonstrate if arthroscopic approach may be better than the open one, reducing recovery time and allowing a rapid recovery of shoulder functionality, with fast return to sport.
**Materials and methods** From January 2001 to March 2010, 46 professional athletes underwent surgical treatment for post traumatic shoulder instability. Two groups were made randomly. They are homogeneous for age, sex and general health conditions. In the first were included 25 athletes treated arthroscopically (Group A) and in the second 21 athletes treated with open approach (Group B). The two surgical treatment groups consist of a capsulolabral reconstruction with anchors. Patients were followed-up from 36 to 92 months, (mean follow-up 64 months) according to Rowe Score for Instability and V.A.S. scoring system.
**Results** Group A showed an excellent joint function recovery, (average Rowe score about 95 pts and average V.A.S. score about 2.5 pts), with full return to sport. Group B showed good clinical results (average Rowe score about 90 pts and average V.A.S. score about 4 pts.), but they returned later to sport (on average 30–45 days later).

There were no nervous or vascular complications in either group and there was only one recurrence in the Group B.
**Discussion** In top level athletes, surgical shoulder repair is indicated even after the first episode of dislocation. Nowadays capsuloplasty can be performed arthroscopically. Arthroscopic surgery allows comparable results to open techniques, providing a less invasive and faster functional recovery. The indications for arthroscopic treatment of shoulder instability are also continuous pain, constant apprehension and repeated episodes of dislocation.
**Conclusions** Arthroscopic surgical approach appears to be excellent in shoulder instability management, reducing recovery time, allowing a faster return to sport, with less pain, if compared with open surgery.

### *SPE04—RESIDENTS' ORAL COMMUNICATIONS*

### Acromion-clavicular joint injuries our experience and review of the literature

L. Andreani*, I. Castellini, G. Ciapini, P. Battistini, L. Bonini

Ortopedia e Traumatologia I (Pisa, IT)

**Introduction** Acromion-clavicular joint (ACJ) injuries represent nearly half of all athletics shoulder injuries. Stability of this joint depends on the integrity of the acromion-clavicular and coraco-clavicular ligaments. Although the traumatic acromion-clavicular joint separation is a well-known topic, there are different classifications, diagnostic procedures, concepts of intervention and a great variety of implants.
**Materials and methods** In this paper we present a retrospective study of all the patients with acute dislocation of ACJ treated during the last 4 years at the first Orthopaedic Department of the University of Pisa with an overview of the recent literature. We treated twenty-six cases of acute ACJ dissociation with 2 different techniques (tight-rope® system and Hook plate). In this series we present only IV or V ACJ type injuries according to Rockwood's classification. Patients were evaluated pre and post operatively using the University of California Los Angeles (UCLA) and Constant's scoring systems.
**Results** The mean Constant score was 90 for the tight-rope® system group and 75 for the hook plate group. At the final follow-up, most of the patients returned to their pre injury level of activity. Two patients had a breakage of the tight-rope® fixating system without any significant new trauma. Postoperative radiographs confirmed anatomic reduction in 18 patients and residual asymptomatic subluxation in 6 patients.
**Discussion** The choice of the best operative technique for the treatment of the ACJ dislocation is a controversial issue. Comparative studies have reported similar results for conservative and surgical treatment, and meta-analysis failed to find any significant benefit in a single surgical approach. The techniques used by us, provided good results with no loss of reduction except in two cases. The hook plate is useful in fractures-dislocation of ACJ, but this requires another surgery in order to remove the plate. On the other hand, the use of the tight-rope® system is a less invasive technique which does not require a new surgery, but rather a temporary stabilization with 2 k wires is needed.
**Conclusions** On the basis that ACJ injuries are frequent, damage must be identified using the Rockwood classification, the surgical approach for type IV, V, VI must be performed within 3 weeks after injury, and the surgeon has to choose the techniques which he/she knows better and feels much more self-confident with.

### Diagnosis and treatment of Idiopathic Toe-Walking

A. Tirelli*, I. Riccio, F. Gimigliano, A. Tirelli

Seconda Università di Napoli (Naples, IT)

**Introduction** Idiopathic Toe-Walking (ITW) is defined as persistent toe walking in a normal child in the absence of developmental, neurological or neuromuscular conditions. The differential diagnosis is made with cerebral palsy, muscular dystrophies, tethered cord syndrome, diastematomyelia, and other neuromuscular diseases. It is frequently associated with language disorders, autism, learning disabilities and with fine motor, visuomotor and gross motor delays. Usually, these patients start their toe-to-toe gait since the beginning of walking, which occurs at a normal age, and have a positive family history in about 30 % of cases. If left untreated, this pattern of gait has the tendency to persist, and although initially, there is full passive dorsiflexion, an equinus contracture can be developed over time. The conservative treatment of ITW includes physical therapy, bracing, serial casting and type A botulinum toxin injection. If the contracture has already developed, the lengthening of the Achilles tendon is necessary. Surgical treatment is reported to provide good clinical outcomes. The purpose of our study is to identify an easy and objective diagnostic protocol.
**Materials and methods** The diagnostic protocol that we propose is divided into two parts. The first one aim at a differential diagnosis from neuromuscular and mild neurological diseases; it includes past and present medical records, evaluation of muscular enzymes, Gower test, evaluation of tendon reflexes and clonus. The second part evaluates the gait pattern and the muscular and joint impairment through the Ashworth scale, the Selective Motor Control scale, the Physician's rating scale, the goniometric measurement of the ankle range of motion and the baropodometry. We applied this diagnostic protocol to all the children referred to our paediatric orthopaedic clinic who use to walk on their toes without a specific diagnosis after an orthopaedic examination.
**Results** Between January 2011 and February 2012, we collected data about 20 children who used to walk on their toes and found out that 4 children were affected by mild diplegia, 4 by childhood transient dystonia and 12 were affected by Idiopathic Toe-Walking.
**Discussion** The diagnosis of idiopathic toe-walking is one of exclusion. We agree with Arvidsson et al. that ITW in childhood is a benign condition. However, we believe it is necessary its early detection in order to avoid the onset of secondary changes in the musculoskeletal system.
**Conclusions** In our experience, the use of a diagnostic protocol helps the paediatric orthopaedic in making a differential diagnosis and choosing the best treatment available.

### Epilepsy and fracture dislocations of the upper limb

G.L. De Pascali*[1], A. Bellu[1], G. De Martinis[1], A. Ferreli[2], N. Coiana[2]

[1]Clinica Ortopedica, Ospedale Marino (Cagliari, IT);
[2]Azienda Ospedaliera "Brotzu" (Cagliari, IT)

**Introduction** Fractures have a larger incidence in epileptic patients because epilepsy's drugs model bone structure [1]. Literature does not describe the incidence of fractures in epileptics who do not undergo therapy. In our study (from 2000 to 2010), with the Neurology Department, we examined 12 epileptics and found 13 fractures; 5 of these were after the first seizure, 12 out of 13 involved the upper limb, 4 were posterior shoulder dislocations; 5 were non-traumatic seizures.

**Materials and methods** Our study is based on admissions at the Neurology Department from 2000 to 2010; it includes epileptic patients who had fracture-dislocation (12 patients). We observed one fracture of the proximal femur and 12 of the upper limb; 4 of these were shoulder fracture-dislocations. All injuries occurred while sleeping.
**Discussion** The increased rate of fractures in epileptics undergoing treatment with anti-convulsant drugs is described in the literature; drugs remodelling bones with increased rate of fractures. The highest increase was for fractures of lower limb (increased rate 9.9); in the upper limb the increased rate was 4.26 [1]. A study by Finelli and others of 1989 [2] states that, given a number of epileptic patients with fracture dislocation, if we consider the fractures in the absence of trauma, 6 out of 9 are fractures dislocations of the neck of humerus. Among these the bilateral fracture posterior dislocation of the shoulder is more common, so it would be pathognomonic of epilepsy because of its low incidence among the non-epileptics. Our case differs from the literature regarding the distribution of fractures (we report upper limb-lower limb ratio of 12:1), but if the data are based on the upper limb injuries we have a prevalence of fracture posterior dislocation of the shoulder as described by Finelli.
**Conclusions** Although there is evidence of the increased rate of fractures in the lower limb compared to the upper limb, we can say than the fracture posterior dislocation of the shoulder, rare in the general population, is pathognomonic of seizure when it is bilateral and tend to suggest the same when unilateral.
**References**
1. Desai KB, Ribbans WJ, Taylor GJ (1996) Injury
2. Finelli PF, Cardi JK (1989) Neurology

### Fractures of the proximal humerus: a comparison between intramedullary nails and interlocking system plates

T. Scuccimarra*, A. Pantalone, D. Vanni, G. Allevi, B. Flacco, M. Berloco, V. Salini

Clinica Ortopedica e Traumatologica, Università "G. d'Annunzio" (Chieti, IT)

**Introduction** Fractures of the proximal humerus represent 3–5 % of all fractures. They often occur as a result of accidental falls, frequently in elderly patients, but can also be caused by high-energy trauma, especially in polytraumatic patients.
**Materials and methods** We conducted a retrospective study of 40 patients undergoing surgery for fracture of the proximal humerus in three fragments, aged between 30 and 75 years. From May 2011 to December 2011 20 patients (group A) were subjected to fixation with intramedullary nail and 20 patients (group B) underwent a osteosynthesis by interlocking system plate. Patients were randomly included in the 2 groups, homogeneous for age, sex and medical condition. The clinical (Constant-Murley) follow-up and X-rays were done at 1, 3 and 6 post-operative months.
**Results** There were no statistically significant differences between the 2 groups (Constant-Murley average of 17 and 15 respectively in group A and B). In the patients included in the first group, less satisfactory reductions of fractures were obtained, in some cases associated with impingement, with the need to remove the nail. However, in group B there were more intraoperative blood losses and 2 cases of avascular necrosis at remote control.
**Discussion** The intramedullary nailing allows to preserve the periosteal circulation, with reduction of intra and post-operative bleeding and hospital stay. In case of displaced not reducible by closed reduction fracture, an open reduction with plate and screws is better.
**Conclusions** The treatment of three fragments-fractures of the proximal humerus is not only a surgical solution. Opt for osteosynthesis with an intramedullary nail or interlocking system plate depends on the degree of displacement of the bone fragments, on the general conditions of the patient and on his functional request, as well as on the preference of the surgeon.

### *SPE02—RESIDENTS' ORAL COMMUNICATIONS*

### Multidisciplinary management in pelvic trauma

G. Topa[1], P. Ditto*[2], G. Sileci[3], P. Vazzana[3], M. Accardo[2], S. Barreca[2], D. Pontoriero[2], M.A. Rosa[2]

[1]UO di Ortopedia e Traumatologia, Azienda Ospedaliera Bianchi-Melacrino-Morelli (Reggio Calabria, IT);
[2]Scuola di Specializzazione in Ortopedia e Traumatologia, Sezione di Ortopedia e Traumatologia, Dipartimento Specialità Chirurgiche, Università degli Studi di Messina (Messina, IT);
[3]Scuola di Specializzazione in Anestesia e Rianimazione, Università degli Studi di Messina (Messina, IT);

**Introduction** The polytrauma is associated with two or more wounded body areas, with any possible impairment of respiratory function and/or circulatory conditions. The mortality of these patients is very high and is the leading cause of death under 40 years: 50 % in the first minutes after the trauma (rupture of the heart, tearing of the brainstem), 30 % during the Golden Hour (hemo-pneumothorax, shock-hemorrhagic, ruptured liver or spleen), 20 % some days/weeks later (Sepsis, M.O.F., CID). The traumas of the pelvis are the major part of high Energy traumas: traffic accidents, falls from heights and crush injuries and 75 % are polytraumas.
**Materials and methods** The authors reviewed their series consisting of 67 cases treated at the Policlinic University G. Martino of Messina and the Ospedale Bianco-Melacrino-Morelli of Reggio Calabria, between 1/1/2007 and 31/12/2011, the age is between 21 and 85 years with a male/female ratio of 2/1. 59 were treated surgically, ORIF-EF; 8 with immobilization in bed. The anesthetic problems were studied and evaluated with both Glasgow Coma Scale and with ASA guidelines. Tile and AO Classifications were employed. Patients were studied both through standard XRay (when possible inlet/outlet) and with 3D reconstruction CT.
**Results** The objectives set by authors were: (1) stabilize the patient's vital signs (ABC); (2) restore bony anatomy; (3) preventing and resolving instabilities and skeletal complications; (4) minimize the possible discomfort for the patient; (5) facilitate the return to the previous life.
**Discussion** Our series includes: 8 cases with immobilization in bed (TILE-A), 22 with anterior (ilioinguinal) approach in the case of disjunctions, complete or incomplete, of SIAS, iliac wing fractures, fractures of the acetabulum; 11 with Pfannenstiel's approach in diastasis of the symphysis; 15 with posterior approach because of the sacroiliac full disjunctions. In Emergency a pelvic C-clamp has been used for rapid reduction and stabilization of the pelvis and for bleeding control. In ASA 3–4 patients the golden treatment was the external fixation (11).
**Conclusions** According to Authors the golden technique is always ORIF, as it allows an anatomical synthesis, even if there is a greater risk of infection, functional recovery is more rapid. Non-operative treatment is reserved to Tile-A fractures, while the treatment of urgency/emergency is always the EF.

### Traumatic triangular fibrocartilage complex tears in the athlete: results after arthroscopic treatment

L. Armillotta*[1], F. Pogliacomi[2], M. Corradi[3], F. Ceccarelli[2]

[1]Scuola Specializzazione in Ortopedia e Traumatologia, Università di Parma (Parma, IT);
[2]Sezione di Ortopedia, Traumatologia e Riabilitazione Funzionale, Dipartimento di Scienze Chirurgiche, Università di Parma (Parma, IT);
[3]U.O. Clinica Ortopedica, Az. Ospedaliero-Universitaria di Parma (Parma, IT)

**Introduction** Traumatic triangular fibrocartilage complex tears are typical of contact sports and the mechanism is commonly a twisting/rotational wrist trauma. These ruptures, which are common causes of ulnar-sided wrist pain, may be particularly disabling. There are only a few general discussions in the literature about this topic related to sport. Treatment options of TFCC injuries vary along a wide spectrum based on acuteness and location of the tear as well as associated osseous and capsular-ligamentous lesions. The aim of this study was to define the correct approach in the treatment of these lesions in competitive athletes who require a high level of wrist function for performance and to evaluate the results of arthroscopic treatment in this type of patients.

**Materials and methods** Between January 2006 and December 2010, 10 athletes, who responded poorly to non-operative treatment, underwent wrist arthroscopy. Repair or debridement was selected according to the location of the rupture, which was divided using the Palmer classification. After surgery all patients underwent 6 weeks of immobilization and similar rehabilitation protocol. Pre-surgery and follow-up Modified Mayo Wrist Score and Patient Rated Wrist/Hand Evaluation were recorded.

**Results** Ruptures were repaired in 7 patients and debrided in 3. Both scores improved in all cases thus allowing return to sport after a mean period of 3.5 months from surgery. In 1 patient with an associated positive ulnar variance and initial chondromalacia of the ulnar head return to play was delayed to 8 months.

**Discussion** Arthroscopic treatment of traumatic fibrocartilage complex injuries allowed return to athletic competition at a pre-injury level in all cases. Return to sport may be influenced or delayed in athletes with associated ulnar sided osseous and/or capsular-ligamentous wrist conditions.

**Conclusions** In high-level athletes arthroscopic surgery has to be considered the treatment of choice. A more aggressive postoperative rehabilitation protocol could be performed after debridement alone, thus allowing an earlier return to sport.
